# miR‐106b‐5p protects against drug‐induced liver injury by targeting vimentin to stimulate liver regeneration

**DOI:** 10.1002/mco2.692

**Published:** 2024-08-21

**Authors:** Xiaoyan Lu, Lingqi Yu, Jie Zheng, Anyao Li, Junying Li, He Lou, Wentao Zhang, Hui Guo, Yuzhen Wang, Xuemei Li, Yue Gao, Xiaohui Fan, Jürgen Borlak

**Affiliations:** ^1^ Pharmaceutical Informatics Institute College of Pharmaceutical Sciences Zhejiang University Hangzhou China; ^2^ State Key Laboratory of Chinese Medicine Modernization Innovation Center of Yangtze River Delta Zhejiang University Jiaxing China; ^3^ State Key Laboratory of Component‐Based Chinese Medicine Tianjin University of Traditional Chinese Medicine Tianjin China; ^4^ Department of Hepatobiliary the First Affiliated Hospital of Tianjin University of Traditional Chinese Medicine Tianjin China; ^5^ Department of Pharmacy Sir Run Run Shaw Hospital Zhejiang University School of Medicine Hangzhou China; ^6^ Department of Pharmaceutical Sciences Beijing Institute of Radiation Medicine Beijing China; ^7^ The Joint‐Laboratory of Clinical Multi‐Omics Research Between Zhejiang University and Ningbo Municipal Hospital of TCM Ningbo Municipal Hospital of TCM Ningbo China; ^8^ Centre for Pharmacology and Toxicology Hannover Medical School Hannover Germany

**Keywords:** adaptive response, drug‐induced liver injury, miRNAs, toosendanin, vimentin

## Abstract

Understanding the endogenous mechanism of adaptive response to drug‐induced liver injury (arDILI) may discover innovative strategies to manage DILI. To gain mechanistic insight into arDILI, we investigated exosomal miRNAs in the adaptive response to toosendanin‐induced liver injury (TILI) of mice. Exosomal miR‐106b‐5p was identified as a specific regulator of arDILI by comprehensive miRNA profiling. Outstandingly, miR‐106b‐5p agomir treatment alleviated TILI and other DILI by inhibiting apoptosis and promoting hepatocyte proliferation. Conversely, antagomir treatments had opposite effects, indicating that miR‐106b‐5p protects mice from liver injury. Injured hepatocytes released miR‐106b‐5p‐enriched exosomes taken up by surrounding hepatocytes. *Vim* (encodes vimentin) was identified as an important target of miR‐106b‐5p by dual luciferase reporter and siRNA assays. Furthermore, single‐cell RNA‐sequencing analysis of toosendanin‐injured mouse liver revealed a cluster of *Vim* ^+^ hepatocytes; nonetheless declined following miR‐106b‐5p cotreatment. More importantly, *Vim* knockout protected mice from acetaminophen poisoning and TILI. In the clinic, serum miR‐106b‐5p expression levels correlated with the severity of DILI. Indeed, liver biopsies of clinical cases exposed to different DILI causing drugs revealed marked vimentin expression among harmed hepatocytes, confirming clinical relevance. Together, we report mechanisms of arDILI whereby miR‐106b‐5p safeguards restorative tissue repair by targeting vimentin.

## Graphical abstract

We showed that miR‐106b‐5p promotes robustly adaptive response to drug‐induced liver injury in animal models of hepatotoxicity. Clinical validation confirms serum miR‐106b‐5p level and its target vimentin to be directly correlated with the severity of liver injury, therefore suggesting that miR‐106b‐5p may be a prognostic biomarker for resolution and has the potential to be exploited as a therapeutic agent.

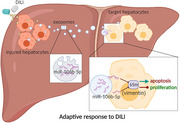

## 1 INTRODUCTION

Many drugs carry the potential to cause drug‐induced liver injury (DILI), leading to severe outcomes such as acute liver failure (ALF), which poses a significant challenge for clinical use and drug development. Although prognostic models for ALF are in use (see MELD score, Kings College criteria, etc.), it is difficult to predict patients who may experience spontaneous remission due to methodological limitations. Additionally, an effective prevention strategy has been halted, because of the complexities underlying the pathogenesis of DILI.

An understanding of adaptive responses to liver injury has attracted much attention for its role in the prevention and treatment of drug‐induced liver disease.^1^ Specifically, the adaptive response is a host defense reaction, which limits tissue injury and restores homeostasis through an endogenous protective mechanism, and has been successfully used in some aspects of clinical practice.^1^, ^2^ For instance, to protect against ischemia–reperfusion injury in liver surgery, ischemic preconditioning has been proposed.^2^ The adaptive response in DILI (arDILI) can be recapitulated at the level of biochemical markers of liver damage which initially rise during drug treatment but return to normal despite continuous medication.^3^ Several hepatotoxic drugs, including isoniazid and acetaminophen (APAP), can elicit adaptive responses.^4^, ^5^ Although an understanding of arDILI is of critical importance in clinical practice with DILI cases, there are no reliable biomarkers to predict arDILI.

Adaptive responses typically involve alterations of toxicity pathways, such as an inhibition and/or induction of CYP450 enzymes,^6^ upregulation of multidrug resistance‐associated proteins, increases in anti‐inflammatory cytokines and cytokine inhibitor production,^7^ induction of antioxidant enzymes,^8^ and upregulation of chaperones.^9^ So far only few studies focused on the mechanisms underlying spontaneous resolution during continuous drug exposure^10^ and adaptation can be regarded as an effective tissue repair process with the regeneration of harmed tissue being orchestrated in a complex manner. In this regard, noncoding RNAs play a decisive role.^11^

microRNAs (miRNAs) are evolutionarily conserved small noncoding RNAs, playing vital roles in physiological and pathophysiological processes including liver regeneration.^11^ It is worth noting that exosomes are extracellular vesicles secreted by a variety of cells^12^ to mediate cell‐to‐cell communication^13^ and miRNAs are important constituents of circulating exosomes and function in the transmission of cellular signals.^14^ Furthermore, miRNAs serve as predictive and prognostic biomarkers that are highly conserved among different species, and circulating exosomal miRNAs carry the potential to reveal mechanisms of liver diseases.^15^, ^16^, ^17^ Likewise, miRNAs are evaluated for their therapeutic potential in the treatment of liver diseases such as chronic viral infection, liver cancer, and nonalcoholic steatohepatitis.^18^, ^19^, ^20^ All the data support the crucial roles of exosomal miRNAs in liver disease and indicate their usefulness in the clinical management of DILI.^21^

Our study aimed to decipher mechanistic events in the adaptive response to DILI by investigating circulating exosomal miRNAs and their target genes. Considering that the majority of studies adopted models such as low‐dose stimulation followed by high‐dose exposure to explore the mechanisms of adaptive response to DILI,^6^, ^9^ we established an animal model with clinical relevance. In this model, we observed adaptive responses to toosendanin (TSN)‐induced liver injury (TILI) during continuous treatment at the same dosage.^22^ Here, using this model, we identified exosomal miR‐106b‐5p as a robust driver in the adaptive response of TILI. The biological function and target gene of miR‐106b‐5p in arDILI were determined in the cell systems, mouse models, and human samples.

## 2 RESULTS

### 2.1 Serum exosomal miR‐106b‐5p is directly related to the development of adaptive responses to TILI

**FIGURE 1:**
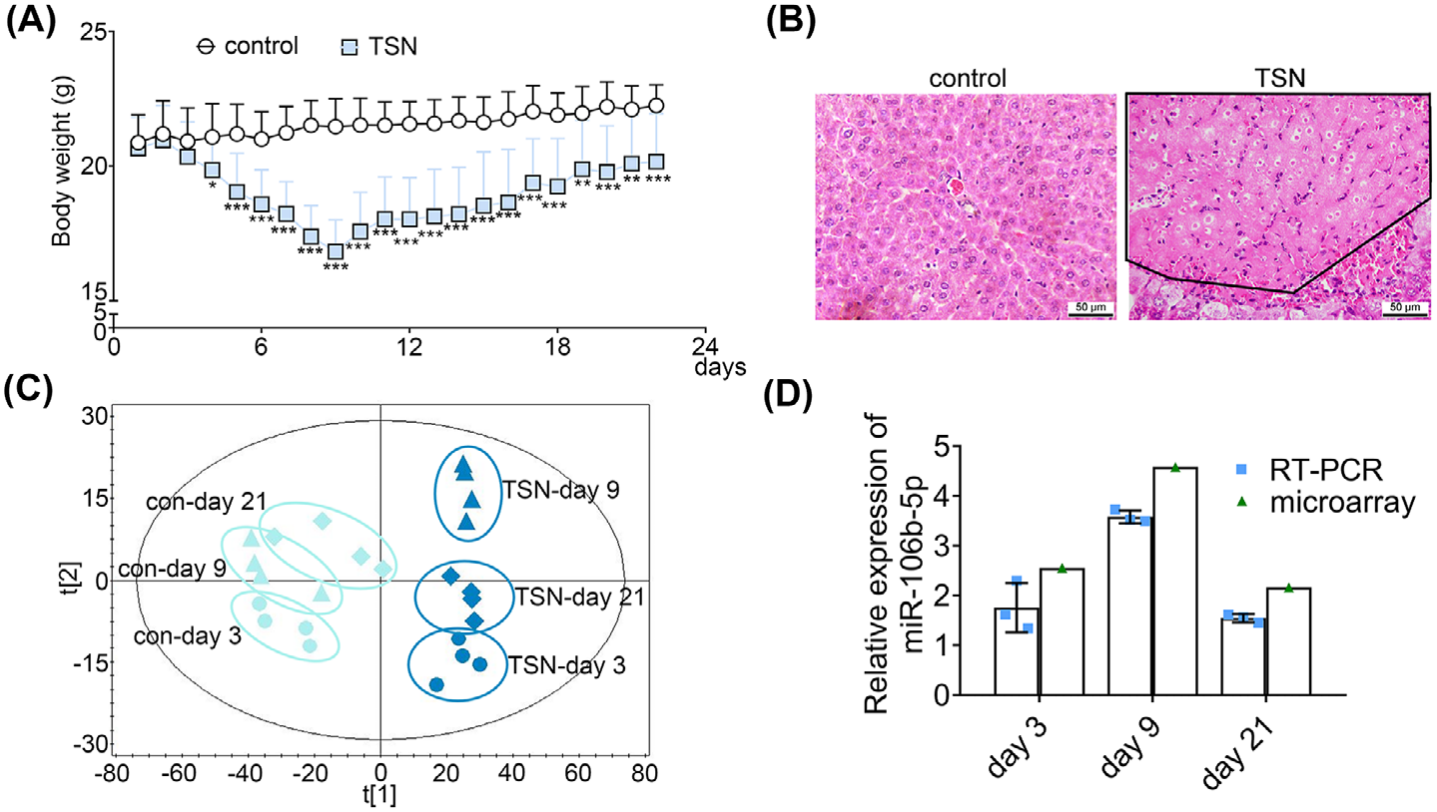
Expression of exosomal miR‐106b‐5p is related to the development of adaptive responses. (A) Body weight versus time. Control group, *n* = 12. TSN group, *n* = 15. The significance was determined using an unpaired Student's *t*‐test. Compared with the control group, ^*^ *p* < 0.05, ^**^ *p* < 0.01, ^***^ *p* < 0.001. (B) Photomicrographs of liver tissues from TILI mice with H&E staining on day 10. The area of hepatic necrosis has been highlighted within the black box. (C) PLS‐DA analysis of serum exosomal miRNA profiles of mice treated with TSN at three time points. (D) Expression levels of serum exosomal miR‐106b‐5p in TSN‐treated mice. Blue: RT‐qPCR results (*n* = 3); white: microarray data. Data are shown as means ± SD.

TSN treatment of mice for 9 days caused significant increases in serum alanine aminotransferase (ALT) and aspartate aminotransferase (AST) while histopathology confirmed hepatic necrosis in liver lobules (Figure 1A,B and Tables S1 and S2). Interestingly, TSN treatment caused a significant 21% reduction in body weight (BW) on day 9 of treatment; however, continuous treatment for 21 days led to partial resolution, with serum markers of liver injury returning to normal and BW significant increasing. These results were consistent with our previous study.^22^

Then, we performed whole genome miRNA expression profiling of serum derived exosomes following TSN and saline treatment of mice on days 3, 9, and 21. Moreover, we assessed the size distribution, morphology, and expression of markers (CD81, TSG101, and syntenin) of the extracted exosomes and confirmed the reliability of serum exosomes (Figure S1).

Next, we performed partial least squares discriminant analysis (PLS‐DA) to differentiate the trajectory of exosomal miRNA profiles. The score plot shows that the TSN‐treatment groups are separate from controls, and we noted a time‐resolved segregation of TSN‐treated animals. A recovery trend is evident among the TSN‐treated groups as the samples of day 9 reached the maximum shift from the control cluster, while on day 21, the TSN‐treated and the control samples are in close proximity (Figure 1C). An analysis of differentially expressed miRNAs (DEMs) revealed 162, 212, and 92 miRNAs as deregulated upon TSN exposure of mice for 3, 9, and 21 days, respectively. We validated the microarray data by real‐time quantitative PCR (RT‐qPCR) and observed good agreement between the two platforms for six randomly selected DEMs (Figure S2). To identify the key miRNAs in the adaptive response of TILI, we performed an ingenuity pathways analysis (IPA). According to the relationship between miRNA and target genes, the DEMs shared in all the top 20 toxic lists of the 3, 9, and 21‐day exposures were selected as candidate miRNAs in the adaptive response of TILI. This revealed miR‐106b‐5p as the only one being commonly induced in all three time points (Table S3). The expression level of miR‐106b‐5p increased with consecutive administrations of TSN for a period of 9 days and subsequently returned to almost control values as evidenced by RT‐qPCR (Figure 1D). We further investigated the expression levels of additional miRNAs in the miR‐17 family, known to be closely associated with cell proliferation and liver regeneration. The results showed that the expression of these other miRNAs in the miR‐17 family was unchanged during the adaptive response of TILI, and only the expression of miR‐17‐5p and miR‐93‐5p increased after 9 days of TSN exposure, but did not change for 3 and 21 days of TSN exposure, which further proved the important role of miR‐106b‐5p in arDILI (Table S4). Based on this finding, we selected miR‐106b‐5p as a potentially key regulator in the adaptive response of TILI.

### 2.2 miR‐106b‐5p protects mice from DILI

**FIGURE 2:**
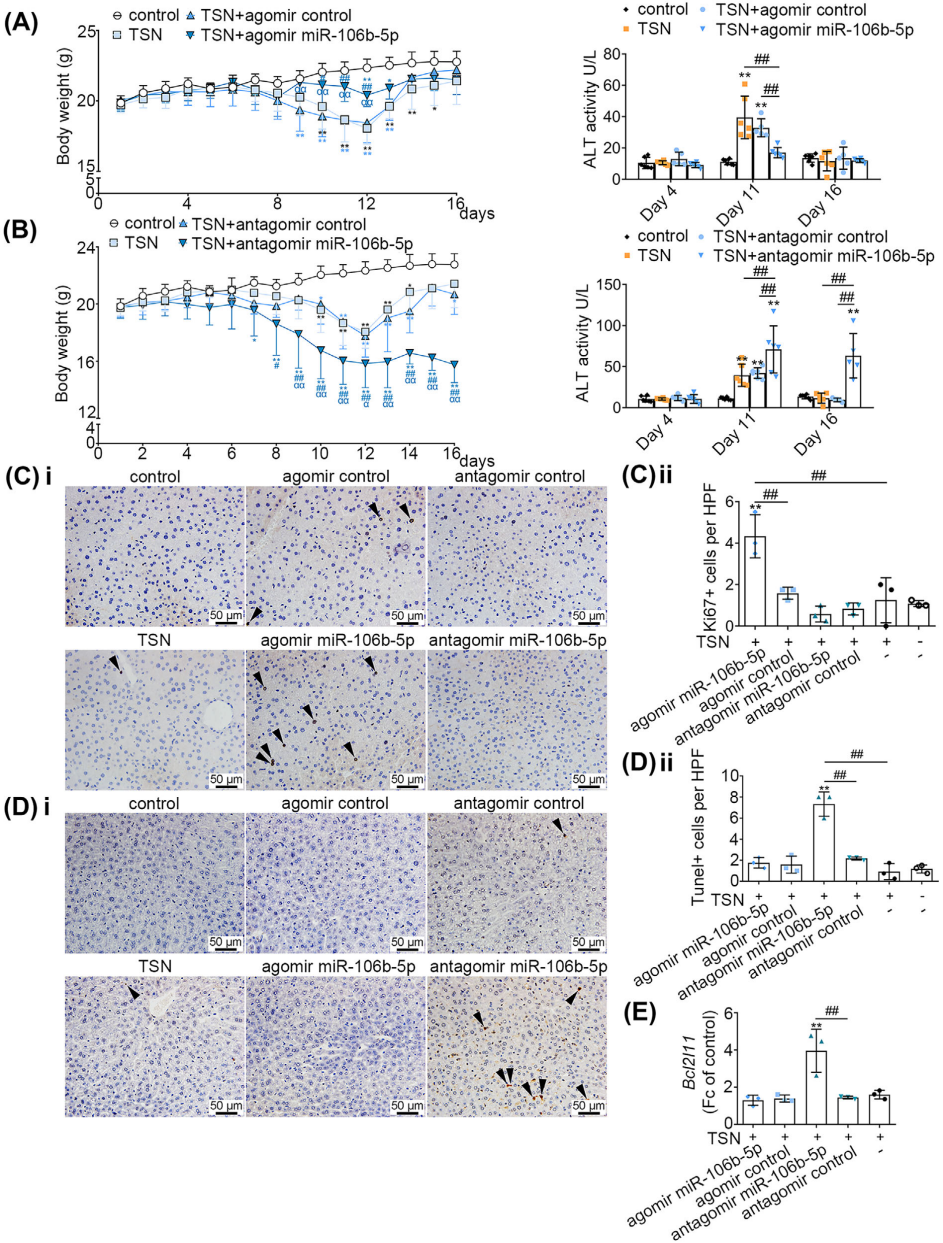
miR‐106b‐5p alleviates DILI in mice. (A) Body weight versus time (left panel), serum ALT activity (right panel). Control group, TSN group, TSN + agomir miR‐106b‐5p group, *n* = 6. TSN + agomir control group, *n* = 5. (B) Antagomir miR‐106b‐5p increased TILI in mice (*n* = 6). Body weight versus time (left panel), serum ALT activity (right panel) in mice (*n* = 6). (C) Ki‐67 immunohistochemical staining of liver sections in TSN‐treated mice on day 16. Representative pictures (400×); brown cells identified by the arrows were Ki‐67 positive cells (panel i). Proportion of Ki‐67 positive cells (panel ii). (D) TUNEL staining of liver sections on day 16. Representative pictures (400×); brown cells identified by the arrows were TUNEL positive cells (panel i). Proportion of brown positive cells (panel ii). Scale bars: 50 µm. (E) The expression level of proapoptotic gene *Bcl2l11* mRNAs in liver sections of mice on day 16 (*n* = 3). The significance was determined with a one‐way ANOVA (A, left panel; B, left panel; C, D, E) or two‐way ANOVA (A, right panel; B, right panel) followed by Tukey's multiple‐comparison test. Compared with the control group, ^*^ *p* < 0.05, ^**^ *p* < 0.01 (A, B, C, D). Compared with the TSN group, ^#^ *p* < 0.05, ^##^ *p* < 0.01 (A, left panel; B, left panel). Compared with the TSN + agomir control (A, left panel) or antagomir control group (B, left panel), ^α^ *p* < 0.05, ^αα^ *p* < 0.01. The composition of the two groups marked with horizontal lines, ^#^ *p* < 0.05, ^##^ *p* < 0.01 (A, right panel; B, right panel; C, D, E).

To define its biological function in TILI, we treated mice with the agomir and antagomir of miR‐106b‐5p as well as appropriate negative controls (NCs). Mice exposed to TSN and the agomir NC developed hepatotoxicity as evidenced by increased serum ALT activities and reduced BW, especially after 11 days of TSN treatment. In strong contrast, mice cotreated with TSN and the miR‐106b‐5p agomir did not develop hepatotoxicity, and serum ALT activities were insignificantly and only mildly changed, when compared with vehicle controls. We obtained similar results when changes in BW were considered (Figure 2A). Therefore, miR‐106b‐5p agomir treatment protected mice from TSN‐induced hepatotoxicity. Meanwhile, the combined treatment of mice with TSN and the miR‐106b‐5p antagomir caused persistent increases in serum ALT activities and a statistically significant reduction in BW (Figure 2B), therefore demonstrating toxicity. Indeed, the antagomir treatment blocked the arDILI response to TSN treatment, and the persistent ALT elevation as well as the reduction in BW on day 16 of TSN treatment are testimony to this effect. Importantly, one animal each died of hepatic failure in the combined TSN and miR‐106b‐5p antagomir as well as the negative antagomir treatment group following 14 days of administration. Overall, the data suggest that miR‐106b‐5p is a key molecule in alleviating TILI by mediating adaptive responses in the liver.

Furthermore, we assessed cell proliferation in TSN and miR‐106b‐5p agomir/antagomir cotreated animals. We observed a significant increase in Ki‐67 positive stained hepatic nuclei in liver sections of the miR‐106b‐5p agomir treatment group; however, this phenomenon was not observed when treated with the miR‐106b‐5p antagomir (Figure 2C). Additionally, we found transferase‐mediated dUTP‐biotin nick end labeling (TUNEL)‐positive cells were significantly increased in the TILI‐injured mice cotreated with the miR‐106b‐5p antagomir. Conversely, miR‐106b‐5p agomir treated animals did not differ in the number of TUNEL‐positive cells when compared with controls (Figure 2D). Consistent with these findings, we observed increased expression of the *Bcl2l11* mRNAs in mice treated with the antagomir of miR‐106b‐5p (Figure 2E). The data indicate that upregulation of miR‐106b‐5p in vivo promotes liver regeneration by stimulating cell proliferation and blocking apoptosis.

To investigate the effect of miR‐106b‐5p on other DILI, APAP‐injured mice were administrated with miR‐106b‐5p agomir. The expressions of miR‐106b‐5p in the serum exosomes and livers of APAP‐induced liver injury mice were also increased compared with the control group, indicating the critical role of miR‐106b‐5p in DILI (Figure S3A). Furthermore, compared with the control mice, serum ALT and AST activities of APAP‐injured mice were significantly increased, whereas miR‐106b‐5p agomir treatment obviously decreased ALT and AST levels (Figure S3B), indicating that miR‐106b‐5p could protect mice from APAP‐induced liver injury.

### 2.3 miR‐106b‐5p stimulates cell proliferation and inhibits apoptosis

**FIGURE 3:**
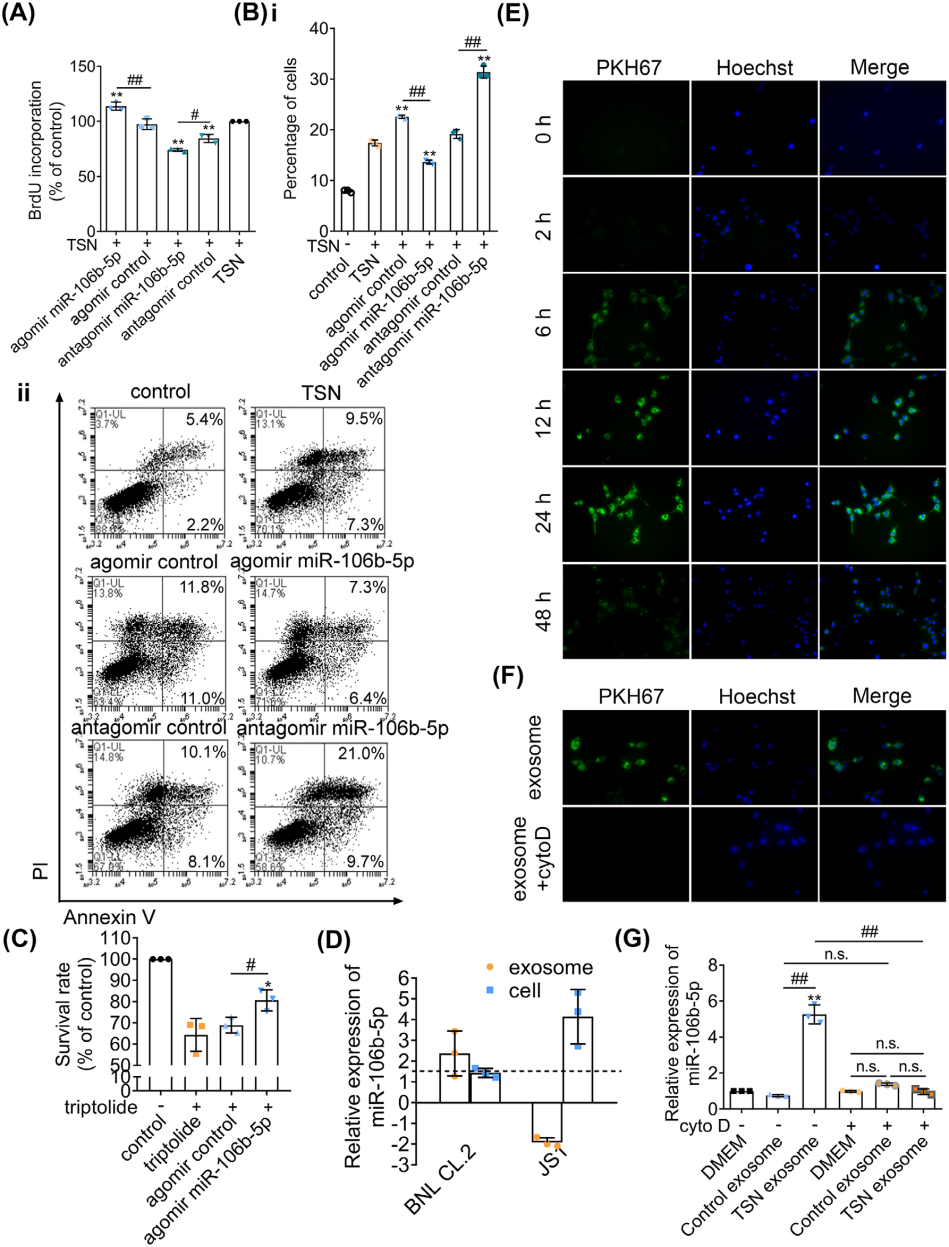
Exosomal miR‐106b‐5p derived from injured hepatocytes and transferred to target hepatocytes. (A) BrdU incorporation rate of BNL CL.2 following 48 h TSN exposure. (B) Annexin V/PI double fluorescence staining of TSN‐injured BNL CL.2. Percentage of annexin V positive cells (panel i). Representative pictures (panel ii). (C) The survival rate of HepG2 after triptolide exposure. (D) Expression of miR‐106b‐5p in cells and supernatant exosomes after TSN exposure. (E) Fluorescence images of BNL CL.2 incubated with 100 µg/mL exosomes at different times. (F) Fluorescence images of BNL CL.2 incubated with 100 µg/mL exosomes in 24 h. (G) The expression of miR‐106b‐5p in BNL CL.2 incubated with different substances (*n* = 3). Statistical differences between the groups were compared with one‐way ANOVA followed by Tukey's multiple‐comparison test. Compared with the TSN group, ^**^ *p* < 0.01 (A and B). Compared with the triptolide group, ^*^ *p* < 0.05 (C). Compared with the DMEM group, ^**^ *p* < 0.01 (G). The composition of the two groups marked with horizontal lines, ^#^ *p* < 0.05, ^##^ *p* < 0.01. n.s., no significance.

Based on the top 20 toxic lists analyzed by IPA of the 3, 9, and 21‐day exposures, the TSN‐treated groups showed a significant enrichment of liver proliferation. Additionally, cell apoptosis‐related pathways were also commonly enriched among all TSN groups at 3, 9, and 21 days, including proapoptosis, liver necrosis/cell death, and p53 signaling (Figure S4). Therefore, “cell proliferation” and “cell apoptosis” were selected as the focus of our subsequent research. To investigate the effect of miR‐106b‐5p on cell proliferation and apoptosis, we cotreated murine BNL CL.2 hepatocyte cell cultures with TSN and agomir or antagomir of miR‐106b‐5p. As shown with the BrdU assay, the agomir treatment stimulated whereas the antagomir treatment inhibited proliferation of TSN‐injured hepatocytes (Figure 3A). Moreover, the agomir treatment inhibited apoptosis while the antagomir treatment had the opposite effect (Figure 3B). Combined with the above TUNEL assay of liver sections, the data support that the notion of miR‐106b‐5p impairs apoptosis and promotes liver regeneration. Additionally, it has been demonstrated that necrosis and apoptosis are interdependent phenomena triggered by the activation of shared pathways and signals in liver injury and diseases,^23^ which was consistent with our results. In addition, compared with that in the agomir control group, the survival rate of triptolide‐injured HepG2 hepatocytes was increased after treatment with miR‐106b‐5p agomir, indicating the protective effect of miR‐106b‐5p against other hepatotoxins (Figure 3C). However, the source of exosomal miR‐106b‐5p and its transmission to target cells requires further investigations.

### 2.4 Hepatocytes are the source of exosomal miR‐106b‐5p

It has been reported that hepatocyte exosomes, but not exosomes from Kupffer cells or sinusoidal endothelial cells, promote hepatocyte proliferation in vitro and liver regeneration in vivo.^24^ Considering the fact that hepatocytes are responsible for tissue restoration and that hepatic stellate cells (HSCs) play an important role in liver regeneration, we hypothesized that hepatocytes or HSCs might be a major source of serum exosomal miR‐106b‐5p. Therefore, we analyzed the expression of miR‐106b‐5p in hepatocytes or HSCs and in exosomes obtained from the culture media after exposure to TSN. Compared with untreated BNL CL.2 hepatocyte cultures, the expression of miR‐106b‐5p in TSN‐injured BNL CL.2 hepatocytes did not differ; however, the expression of miR‐106b‐5p in the exosomes obtained from the TSN‐exposed hepatocyte culture media was significantly increased. In strong contrast and when compared with vehicle controls, the expression of miR‐106b‐5p in JS1 HSCs increased significantly after exposure to TSN but the expression of miR‐106b‐5p in the exosomes of the supernatant decreased (Figure 3D). Moreover, the expression of miR‐106b‐5p in the exosomes obtained from the TSN‐exposed HepaRG hepatocyte culture media was significantly increased, compared with exosomes from untreated HepaRG hepatocyte cultures (Figure S5), indicating that human hepatocytes also released miR‐106b‐5p‐enriched exosomes. These data demonstrate TSN‐injured hepatocytes are the main sources for circulating exosomal miR‐106b‐5p.

Next, we examined the cellular uptake of exosomal miR‐106b‐5p. We harvested exosomes secreted by BNL CL.2 hepatocytes and labeled them with the fluorescent membrane dye PKH67. Following incubations for 6 h, we observed uptake of PKH67‐labeled exosomes by BNL CL.2 cells, and a maximum was reached after 24 h. Therefore, we obtained evidence for the uptake of exosomes by hepatocytes in a time‐dependent manner (Figure 3E). Conversely, cytochalasin D (cyto D) blocked the uptake (Figure 3F).

Furthermore, we added the exosomes secreted by untreated hepatocytes and those secreted by the TSN‐injured hepatocytes to TSN‐exposed BNL CL.2 hepatocyte cultures. Compared with the control group, the expression of miR‐106b‐5p did not differ when exposed to exosomes secreted by untreated hepatocytes. However, the expression of miR‐106b‐5p increased markedly after exposure to exosomes secreted by TSN‐injured hepatocytes. After blocking the uptake of exosomes with cyto D, the expression level of miR‐106b‐5p was not increased when exposed to exosomes secreted by TSN‐exposed hepatocytes (Figure 3G). Collectively, injured hepatocytes release miR‐106b‐5p to alleviate TSN‐induced cytotoxicity.

### 2.5 *Vim* is a direct target of miR‐106b‐5p

**FIGURE 4:**
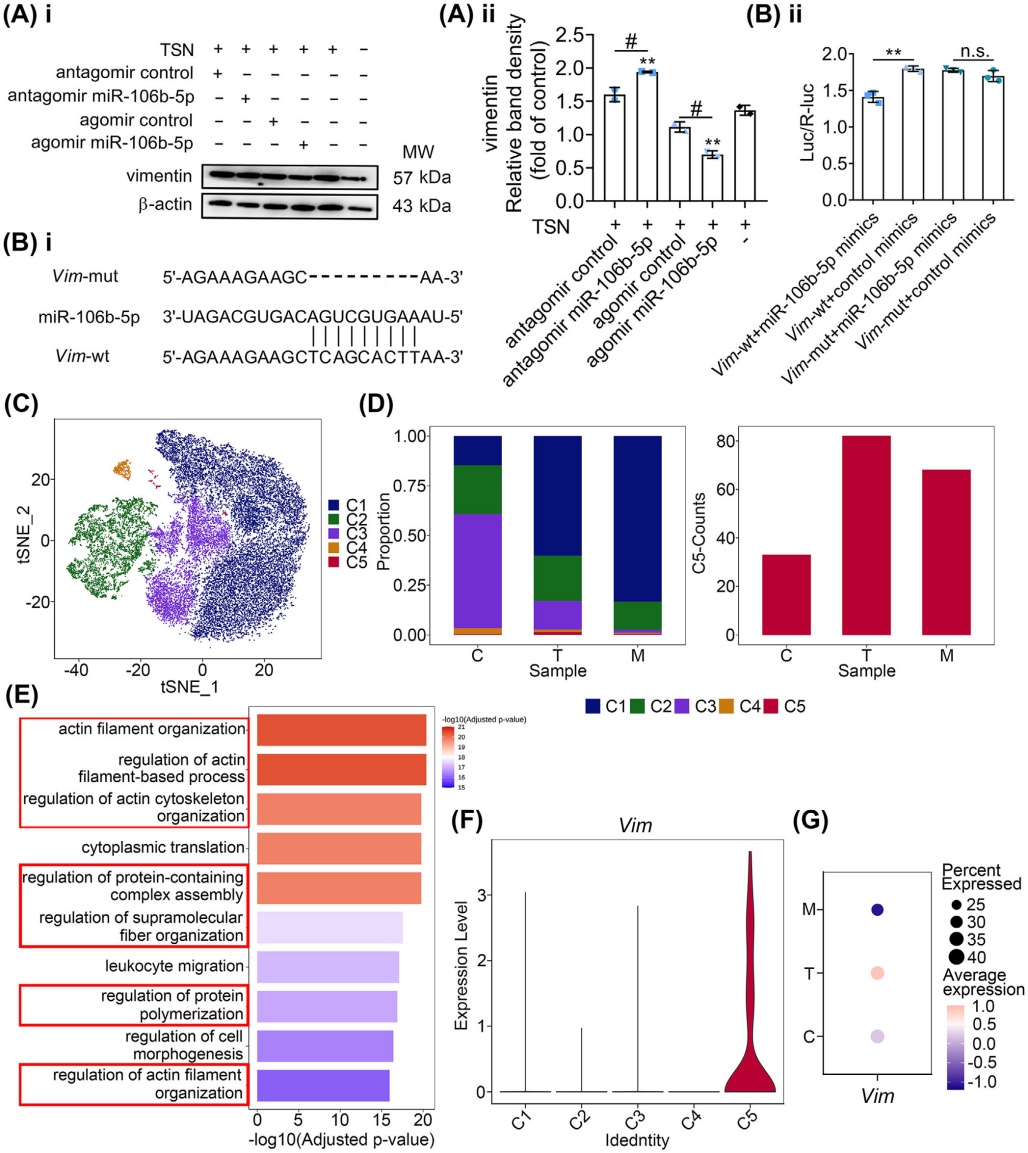
*Vim* is a direct target of miR‐106b‐5p. (A) Vimentin expression in BNL.CL2 cells measured using western blot. Representative pictures (panel i). Quantification of vimentin expression (panel ii). (B) The binding site of *Vim* to miR‐106b‐5p; “wt” indicated wild type, “mut” indicated mutation type, “‐” was a base deletion mutation (panel i). Relative fluorescence intensity of different groups (panel ii). One‐way ANOVA followed by Tukey's multiple‐comparison test was used. Compared with the TSN treatment, ^**^ *p* < 0.01 (A). The composition of the two groups marked with horizontal lines, ^#^ *p* < 0.05 (A), ^**^ *p* < 0.01 (B). n.s., no significance. (C) tSNE plot showing five hepatocyte clusters labeled as C1–C5. (D) Fraction of each hepatocyte subset relative to all hepatocytes (left panel) and cell number (right panel) of each subset. (E) GO enrichment terms of C5. (F) A violin plot showing the expression of *Vim* in each hepatocyte subpopulation. (G) Dot plot showing the expression of *Vim* in C5. Dot size represents the percent expressed cells within C5 subset, and the color refers to the level of average expression.

The effects of miR‐106b‐5p on TSN‐injured hepatocytes prompted us to explore its downstream effectors. Using the “target filter” in the IPA software (Table S5), we identified 44 experimentally validated targets of miR‐106b‐5p. Among them, we focused on the genes that are involved in wound repair, proliferation, and apoptosis, thus *Cdkn1a*, *Ccnd1*, *Vim*, and *Bcl2l11* have been selected for further study. The mRNA expression of *Vim* was significantly reduced in the TSN and miR‐106b‐5p agomir‐treated liver of mice compared with the agomir control group. On the contrary, after the treatment of TSN and miR‐106b‐5p antagomir, the expression of *Vim* mRNA in the liver significantly increased compared with the antagomir control group (Figure S6A). *Ccnd1* displayed similar patterns, whereas *Cdkn1a* and *Bcl2l11* only showed a similar trend under antagomir treatment (Figures S6A and 2E). Furthermore, we transfected BNL CL.2 cells with siRNAs against *Vim* or *Ccnd1*. The viability of cells increased with the silencing of *Vim* determined by the MTT assays, while it decreased with the silencing of *Ccnd1* (Figure S7A). Considering the function of vimentin (the protein encoded by *Vim*) in liver fibrosis (an abnormal wound repair process caused by liver injury)^25^ and the aforementioned results, we focused on the gene *Vim*. Experiments with TSN and miR‐106b‐5p agomir treated BNL CL.2 hepatocytes showed marked reductions in vimentin protein expression (> 30%) compared with the agomir control group (Figure 4A). Consistently, the protein expressions of vimentin in mouse livers showed a similar pattern (Figure S6B).

To verify that *Vim* is a direct target of miR‐106b‐5p, we generated wild‐type and mutant double‐luciferase plasmids according to the predicted binding sites (Figure 4B, panel i). The reporter assay revealed that miR‐106b‐5p significantly reduced the luciferase activity of the wild‐type but not the mutant *Vim* construct, therefore suggesting that *Vim* is a target gene of miR‐106b‐5p (Figure 4B, panel ii).

### 2.6 miR‐106b‐5p inhibits a cluster of *Vim* ^+^ hepatocytes induced by TILI

We obtained in vitro evidence that hepatocytes take up miR‐106b‐5p‐enriched exosomes secreted by damaged hepatocytes and *Vim* is a target gene of miR‐106b‐5p. To confirm its relevance in vivo, we performed single‐cell RNA‐sequencing analysis of hepatocytes in TSN and miR‐106b‐5p agomir cotreated mice following daily dosing for 9 days. After quality filtering, 33,768 hepatocytes were obtained, of which 7992, 5848, and 14,231 cells, respectively were collected from control, TSN, and TSN and miR‐106b‐5p agomir cotreated animals. Unsupervised clustering and t‐distributed stochastic neighbor embedding (t‐SNE) visualization revealed five subclusters, termed C1 to C5, which we identified based on their highly expressed genes (Figures 4C and S7B). Among the five hepatocyte subpopulations, only C5 increased after TSN administration but decreased following miR‐106b‐5p agomir cotreatment. Following TSN treatment, the proportion of the C5 subpopulation increased significantly by a factor of >3, that is, 1.40% (TSN) as compared with 0.41 and 0.48%, respectively in the control and miR‐106b‐5p agomir treatment group (Figure 4D, left panel). We obtained similar results in regards to cell numbers (Figure 4D, right panel). Gene Ontology (GO) enrichment analysis showed regulation of cytoskeleton related biological processes as significantly enriched in the C5 subpopulation (Figure 4E), and vimentin is a key component of the cytoskeleton with important biological functions including cell motility^26^ and division.^27^ However, C1–C4 subpopulations did not enrich pathways related to the regulation of cytoskeleton (Figure S7C–F). In fact, the C5 subset hepatocytes are the only cluster enriched for *Vim* expression (Figure 4F) and the presence of this cluster was confirmed through fluorescence staining of cytokeratin 18 (maker of hepatocytes), Tmsb4x (maker of C5 hepatocytes), and vimentin in the TSN‐treated mouse liver section (Figure S8). Moreover, the dot plot shows that TSN treatment caused strong *Vim* expression in the C5 cluster but was absent in the miR‐106b‐5p agomir treatment group (Figure 4G). Together, the data imply a heterogeneous response to TSN‐induced hepatotoxicity and the results suggested that miR‐106b‐5p and *Vim* are key players in restorative repair.

### 2.7 *Vim* regulates cell proliferation and apoptosis in TSN‐injured BNL CL.2

**FIGURE 5:**
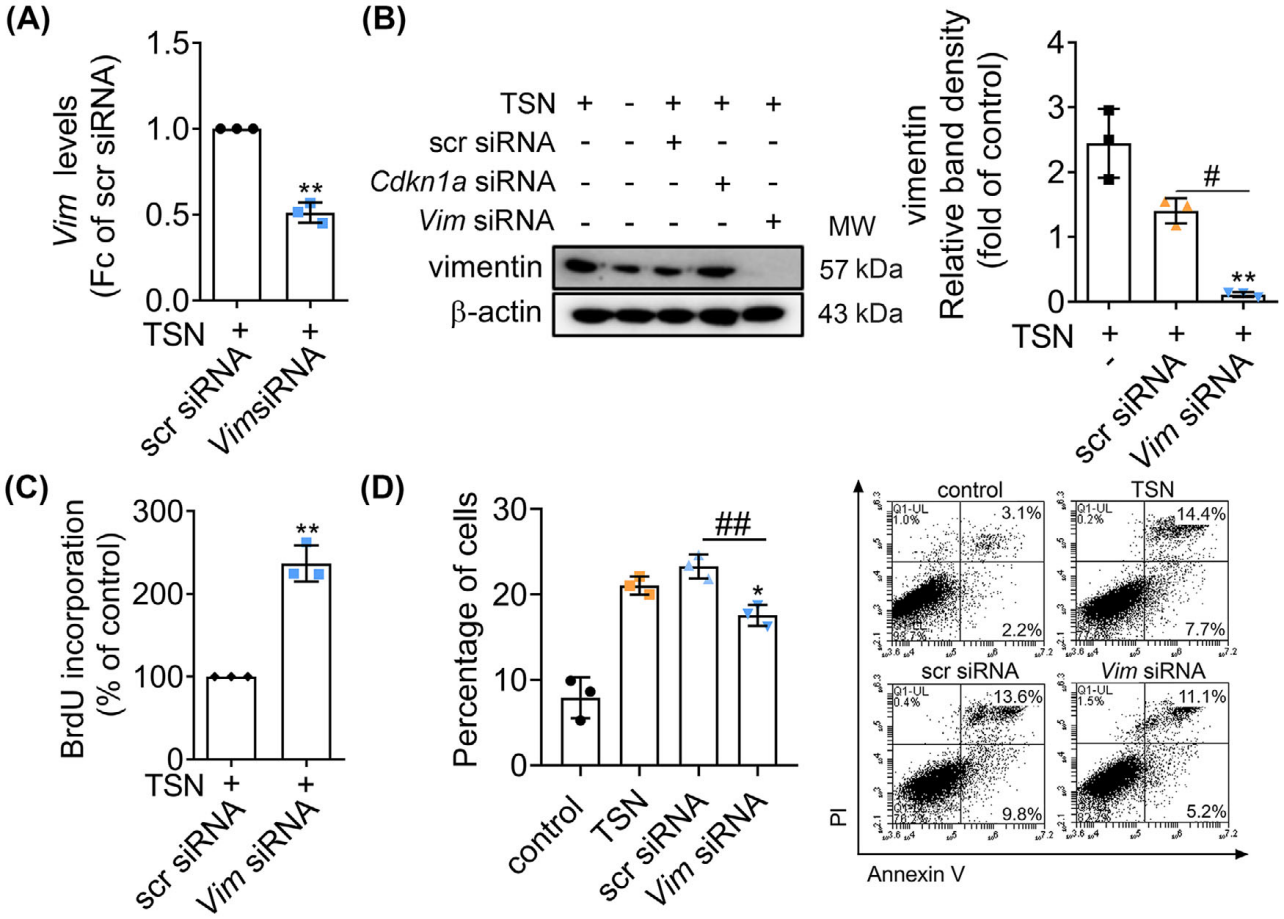
*Vim* modulates proliferation and apoptosis in BNL CL.2 cells. (A) Expression of *Vim* was determined with RT‐qPCR. (B) Expression of vimentin was determined using western blot. (C) Quantification of BrdU incorporation was determined in cells transfected with the *Vim* plasmid along with TSN. (D) Annexin V‐FITC/PI double fluorescence staining of TSN‐injured cells. Representative pictures (left panel). Percentage of annexin V positive cells (right panel). *n* = 3. The significance was determined with a one‐way ANOVA followed by Tukey's multiple‐comparison test (A–C) and an unpaired Student's *t*‐test (D). Compared with the scr siRNA group, ^*^ *p* < 0.05, ^**^ *p* < 0.01 (A and C). Compared with the TSN group, ^*^ *p* < 0.05, ^**^ *p* < 0.01 (B and D). Compared with the scr siRNA group, ^#^ *p* < 0.05, ^##^ *p* < 0.01 (B and D).

To assess the function of *Vim* in TSN‐induced toxicity, we silenced its gene expression by siRNA in BNL CL.2 hepatocytes and such treatment led to a decrease in *Vim* mRNA and vimentin protein levels (Figure 5A,B). The results of the BrdU assay showed that the cell proliferation increased after gene silencing of *Vim* (Figure 5C). Moreover, apoptosis was significantly reduced in BNL CL.2 cells transfected with the *Vim* siRNA (Figure 5D). The results imply that *Vim* is the target gene of miR‐106b‐5p and miR‐106b‐5p targeting *Vim* mitigates TSN‐induced hepatotoxicity by regulating cell proliferation and apoptosis.

### 2.8 *Vim* gene knockdown alleviates TILI and acetaminophen‐induced liver injury (AILI)

It has been reported that lacking vimeintin has no obvious influence on the reproduction and development of mice.^28^ The phenotype of *Vim* knockout (*Vim* ^−/−^) or liver‐specific conditional knockout (*Vim*‐cKO) mice was similar to wild‐type (WT) mice, without significant collagen fiber deposition, necrosis, inflammation, and other lesions, as observed by Masson and H&E staining (Figure S9). Based on the above information, we considered that miR‐106b‐5p mediated processes may not have direct or specific consequences on the liver itself in the clinic. Therefore, we treated *Vim* ^−/−^ and WT mice with TSN for 9 consecutive days. Unlike WT mice, the serum ALT activities and the number of apoptotic cells decreased significantly in *Vim* ^−/−^ mice after TSN exposure (Figure S10A,B). Moreover, cell proliferation of hepatocytes increased in *Vim* ^−/−^ mice (Figure S10C). These results demonstrate that *Vim* knockout alleviates TILI and supports resolution by promoting cell proliferation and inhibiting apoptosis.

**FIGURE 6:**
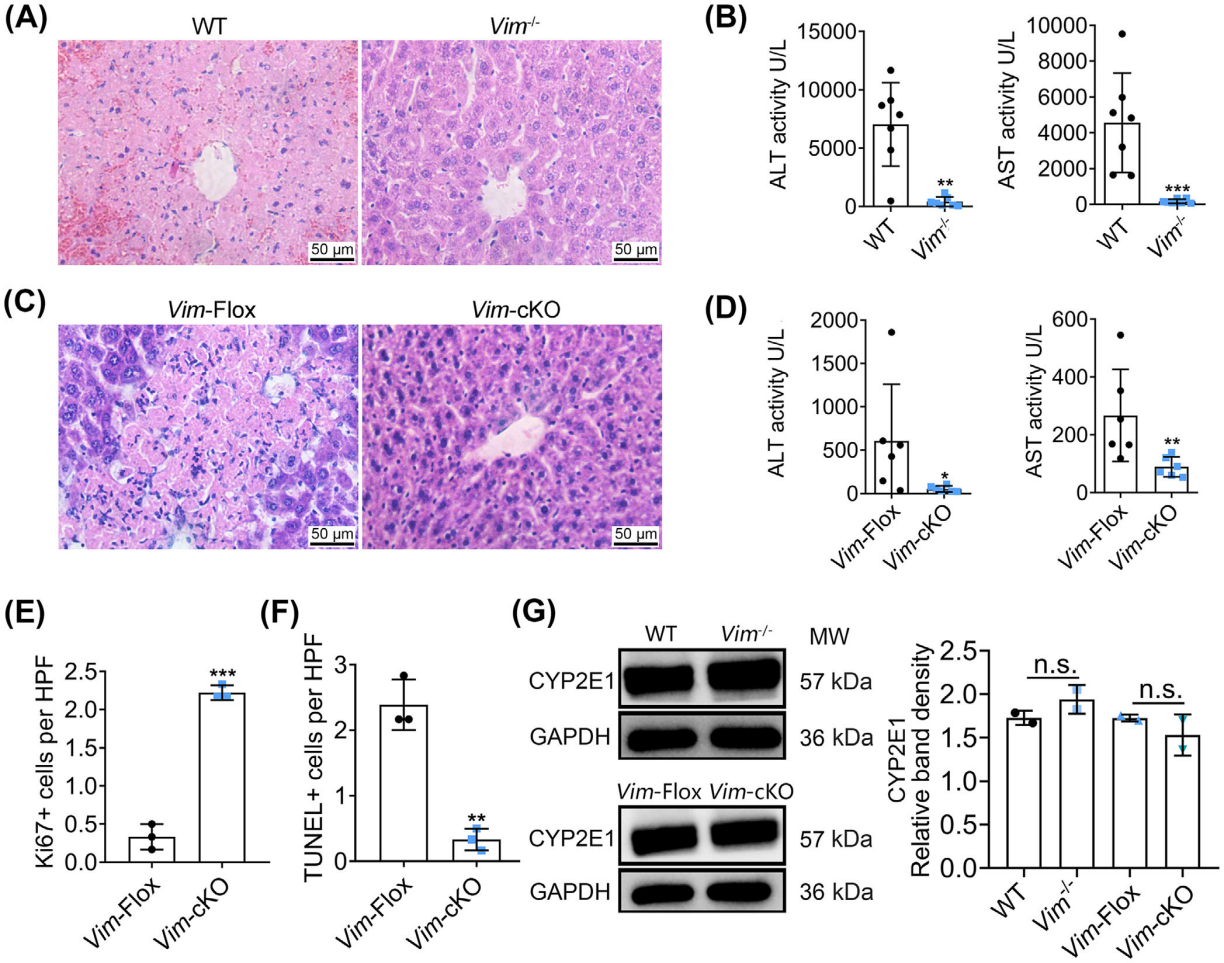
Knockout of *Vim* alleviated APAP‐toxicity in mice. (A) Photomicrographs of liver tissues from AILI mice with H&E staining. The images are representative of at least three independent sections. (B) Serum ALT and AST activities in mice treated with APAP (*n* = 7). (C) Photomicrographs of liver tissues from AILI mice with H&E staining. Magnification: 400×. The scale bar is 50 µm. The images are representative of at least three independent sections. (D) Serum ALT and AST activities in *Vim*‐cKO mice exposure to APAP (*n* = 6). (E) Proportion of Ki‐67 positive cells in AILI mice. (F) Proportion of TUNEL positive cells in AILI mice. (G) Expression of CYP2E1 was determined with western blot. Representative pictures (left panel). Quantification of CYP2E1 expression (right panel). The significance was determined by unpaired Mann–Whitney *U* test (B and D) or two‐tailed unpaired Student's *t*‐test (E, F, G). Compared with the WT group, ^**^ *p* < 0.01, ^***^ *p* < 0.001 (B). Compared with the *Vim*‐Flox group, ^*^ *p* < 0.05, ^**^ *p* < 0.01, ^***^ *p* < 0.001 (D, E, F).

Next, we assessed whether the knockdown of *Vim* would be effective with other agents known to cause liver injury. We chose APAP, that is, a well‐known DILI causing agent. *Vim* ^−/−^ and WT mice received a single intraperitoneal (i.p.) dose of 300 mg/kg APAP. The APAP treatment caused liver injury in WT mice, as indicated by extensive hepatocyte necrosis and intrahepatic hemorrhage and marked changes in serum transaminases. In strong contrast, the livers of *Vim* ^−/−^ mice exhibited almost normal histology (Figure 6A) and serum ALT and AST activities were significantly decreased (Figure 6B). To define the role of *Vim* in APAP‐induced hepatotoxicity, we additionally examined liver‐specific *Vim*‐cKO mice and littermate *Cre* ^−^ control mice (*Vim*‐Flox). The APAP treatment caused hepatocyte necrosis in floxed mice but slight damage in *Vim*‐cKO mice (Figure 6C). Again, the levels of serum ALT and AST were markedly reduced in *Vim*‐cKO mice (Figure 6D). Moreover, Ki‐67 staining indicated increased proliferation of hepatocytes in the livers of *Vim*‐cKO mice after APAP treatment (Figures 6E and S10D), while the results of TUNEL assay showed the number of apoptotic cells to be decreased (Figures 6F and S10E). To exclude the possibility that vimentin knockout mice are unable to metabolize APAP and thus are resistant to injury, we examined the expression of CYP2E1 in the livers of *Vim* ^−/−^ and *Vim*‐cKO mice after APAP treatment. We found CYP2E1 to be abundantly expressed in *Vim* knockout mice and therefore exclude the possibility that the absence of the key enzyme for the production of the toxic N‐acetyl‐p‐benzoquinone imine metabolite protected *Vim* knockout mice from APAP toxicity (Figure 6G). Collectively, these results suggested *Vim* knockout mice are protected from APAP‐induced hepatotoxicity with increased cell proliferation and reduced apoptosis.

### 2.9 miR‐106b‐5p and its target vimentin are related to clinical DILI

**FIGURE 7:**
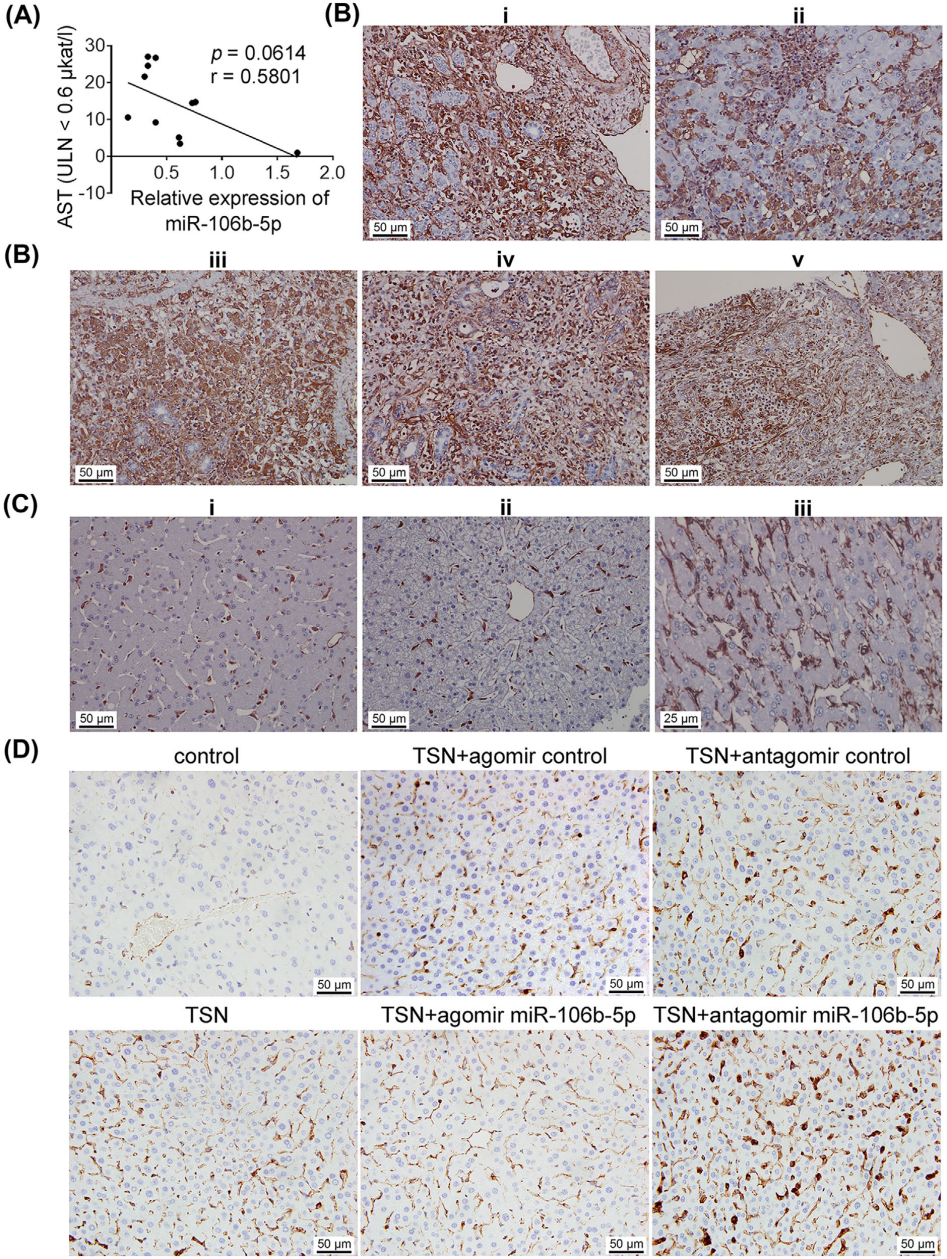
Regulation of serum miR‐106b‐5p and its target vimentin in clinical DILI cases. (A) Serum miR‐106b‐5p expression levels among patients diagnosed with severe DILI (*n* = 11). Serum miR‐106b‐5p expression levels are negatively correlated with AST activities. Data were analyzed by Pearson correlation coefficient. The relationship is borderline significant (*p* = 0.0614). (B) Immunostaining of vimentin in severe clinical DILI cases. Detailed information of patients was listed in Table S6. Marked vimentin expression was detected on harmed hepatocytes, inflammatory cell infiltrates and Kupffer cells. (C) Immunohistochemical staining of vimentin in the liver sections from patients without DILI. Liver biopsy of a female donor aged 51−60 years with no prior history of liver disease. Unlike hepatocytes, Kupffer cells express vimentin (panel i). Liver biopsy of a male donor aged 51−60 years with no prior history of liver disease. Hepatocytes do not express vimentin but Kupffer cells do (panel ii). Liver biopsy of a 31−40 year old female post mortem case with no prior history of liver disease (panel iii). Hepatocytes do not express vimentin; Kupffer cells and sinusoidal endothelium are vimentin positive. (D) Immunohistochemical staining of vimentin in liver sections of mice cotreated with TSN and the agomir or antagomir miR‐106b‐5p on day 16.

To examine the role of miR‐106b‐5p and its target vimentin among clinical cases, we first assayed serum miR‐106b‐5p and AST transaminase activity in patients diagnosed with severe DILI. As shown in Figure 7A, serum miR‐106b‐5p expression levels are negatively correlated with AST activities. In other words, low miR‐106b‐5p levels are associated with high AST activities and therefore severe injury whereas resolving patients presented increased miR‐106b‐5p expression levels with reduced AST transaminase activities. Importantly, miR‐106b‐5p expression levels differed between admission and resolution by a factor of >10‐fold within patients, and the results agree with findings obtained from animal studies where expression levels of miR‐106b‐5p also increased during the resolution phase.

In a separate cohort, we show vimentin protein expression markedly increased in hepatocytes, inflammatory cell infiltrates, and Kupffer cells of patients diagnosed with ALF linked to diclofenac, phenprocoumon, and herbal‐induced liver injury (Figure 7B). A brief description of these cases is given in the figure caption and Table S6.

It is of considerable importance that vimentin is not expressed in healthy hepatocytes (Figure 7C) and independent investigators confirm this notion.^29^, ^30^ Similarly, vimentin is not expressed in hepatocytes of control mice, but in harmed hepatocytes of TSN and agomir or antagomir NC cotreated animals. Obviously, the number of hepatocytes expressing vimentin increased following TSN and the antagomir cotreatment. Conversely, the combined TSN and miR‐106b‐5p agomir treatment prevented vimentin expression (Figures 7D and S11). These results demonstrated that vimentin is not expressed in healthy hepatocytes but in harmed hepatocytes in clinic.

Together, we report experimental and clinical data that highly confirm the clinical relevance of serum miR‐106b‐5p and vimentin in DILI.

## 3 DISCUSSION

An understanding of adaptive responses to drugs with liver liabilities is of great importance, particularly for those where no alternative medication exists and continuous exposure is anticipated. There is an unmet need to better comprehend the mechanisms of arDILI and to define prognostic and predictive biomarkers. Such knowledge will offer new directions in the prevention and management of clinical DILI. Here, we report a novel mechanism of adaptive responses in DILI whereby miR‐106b‐5p safeguards restorative tissue repair.

Recently, miR‐106b‐5p emerged as a hot topic in research; its oncogenic function was demonstrated in diverse human cancers by promoting cancer cell migration and invasion.^31^ However, some studies have suggested its tumor suppressor properties in breast cancer, epithelial ovarian cancer, and papillary thyroid cancer, while also indicated its controversial role in colorectal cancer and non‐small cell lung cancer, where it acts as both a tumor promoter and suppressor, reflecting tissue‐specific and spatial‐specific patterns.^31^ Moreover, the role of miR‐106b‐5p in the pathophysiology of DILI is poorly understood. We demonstrate miR‐106b‐5p to serve as a novel biomarker for arDILI. By administering a miR‐106b‐5p mimic or its inhibitor to TSN‐exposed mice, we confirmed upregulation of miR‐106b‐5p to be a key event of arDILI. The protection effects of miR‐106b‐5p against triptolide‐, TSN‐ or APAP‐induced liver injury were verified. Although studies on the role of miR‐106 in tissue repair are still scarce, an independent research evidenced the miR‐106b∼25 cluster to accelerate hepatocyte proliferation in rats after partial hepatectomy.^32^ A similar effect is seen with the oncomir miR‐21.^33^ This suggests that oncogenic miRNAs also stipulate proliferative effects in response to injury in nonmalignant conditions. Besides, intravenous (i.v.) administration of miR‐106b‐5p and miR‐222‐3p promoted post‐injury β‐cell proliferation in a mouse model of insulin‐deficient diabetes.^34^ Collectively, the published data combined with the findings of the present study shed light on a new paradigm whereby miR‐106b‐5p promotes tissue repair in different organs apart from its oncogenic function.

Our in vitro studies showed that hepatocytes represent the main source of exosomal miR‐106b‐5p. In an effort to augment repair, exosomes released from injured hepatocytes deliver miR‐106b‐5p to other hepatocytes. This concept is supported by the following observations. First, the expression level of miR‐106b‐5p increased significantly in exosomes secreted by TSN‐injured hepatocytes and the exosomes are taken‐up by hepatocytes. Second, the expression level of miR‐106b‐5p in hepatocyte cultures is markedly increased after incubation with exosomes secreted by TSN‐injured hepatocytes; however, it does not change after incubation with exosomes secreted by healthy hepatocytes. Third, after blocking cellular uptake of exosomes by cyto D, the expression level of miR‐106b‐5p did not increase following incubation of exosomes secreted by vehicle or TSN‐exposed hepatocyte cultures (Figure 3F). Notably, exosomes from hepatocytes, but not from other resident cells of the liver, transfer neutral ceramidase and sphingosine kinase 2 to surrounding hepatocytes to induce dose‐dependent hepatocyte proliferation.^24^ HSCs were not the main source of exosomal miR‐106b‐5p as the expression level of miR‐106b‐5p in exosomes released from HSCs was not increased following TSN treatment. Obviously, DILI and its resolution are complex biological processes mediated by various cells^35^, ^36^, ^37^ and further studies are needed to examine interactions of exosomes with target cells in arDILI.

Mechanistically, we delineate a previously unrecognized pathway showing that miR‐106b‐5p promotes liver proliferation and inhibits cell apoptosis by targeting *Vim* in the adaptive response of TILI. Gene knockdown of vimentin to alleviate TSN‐ and APAP‐induced hepatotoxicity and provide experimental evidence for miR‐106b‐5p to target vimentin and to suppress its expression. Studies reported that vimentin plays a key role in wound healing.^38^ One report suggests vimentin‐deficient adult and embryonic mice to display impaired wound healing, though these mice are phenotypically normal.^39^ On the other hand, corneas are protected from traumatic alkali‐induced fibrosis with regenerative healing being promoted in *Vim* ^−/−^ mice.^40^ Vimentin is critically involved in liver fibrosis with the scarring of the liver representing defective wound repair. Moreover, vimentin is a mesenchymal marker for epithelial–mesenchymal transition (EMT).^25^ Whether hepatocytes undergo EMT is controversial;^30^ however studies have suggested that inhibition of EMT alleviated carbon tetrachloride‐induced liver injury,^41^ which was consistent with our findings.

We performed liver single‐cell RNA‐sequencing analysis of TSN‐treated mice and identified a subcluster of *Vim^+^* hepatocytes. Importantly, the number of *Vim^+^* hepatocytes decreased following miR‐106b‐5p agomir cotreatment. This analysis revealed a heterogeneous response in the liver of TSN exposed mice and subcluster C5 in TSN group represented the severely harmed hepatocytes with marked expression of *Vim*. Additionally, we show *Vim* knockout mice protected from TSN and APAP‐induced liver injury and demonstrate in clinical DILI cases the importance of vimentin. So far, only a few studies focused on the functional role of vimentin in DILI; its reduced expression was reported after treatment of mice with the hepatoprotective agent dioscin.^42^ For the first time, we demonstrate miR‐106b‐5p to be a key player in arDILI, and vimentin is expressed in harmed hepatocytes of mice and clinical DILI cases but not in hepatocytes of normal livers. Therefore, vimentin signifies defective wound repair. Collectively, miR‐106b‐5p supports liver repair by targeting *Vim* and protects harmed hepatocytes from defective wound repair in response to DILI. Consistent with our findings for the liver, an inhibition of EMT in tubular epithelial cells restored the proliferative and regenerative capacity of the kidney in mice.^43^

In summary, we demonstrate miR‐106b‐5p is a robust promoter in the adaptive response of DILI and *Vim* is a direct target of it. The knowledge gained from this study advances our understanding of the role of miR‐106b‐5p in DILI. Clinical validation confirms the expression of serum miR‐106b‐5p and its target vimentin to be correlated with the severity of liver injury, therefore suggesting that miR‐106b‐5p is a solid prognostic biomarker for resolution and has the potential to be exploited as a therapeutic agent.

## 4 MATERIALS AND METHODS

### 4.1 Animals

All animal experimental protocols were approved by the Animal Care and Use Committee of the Zhejiang University School of Medicine. We obtained male C57BL/6J and BALB/c WT mice (6–8 weeks old) from the Shanghai SLAC Laboratory Animal Co., Ltd and the Beijing Vital River Laboratory Animal Technologies Co., Ltd. BALB/c *Vim* ^−/−^ mice and liver‐specific *Vim*‐cKO C57BL/6J mice as well as *Vim*‐Flox littermates were derived from GemPharmatech Co., Ltd. (China). Both BALB/c and C57BL/6J mice are inbred strain mice that are commonly used in hepatotoxicity research.^44^, ^45^ Due to the liver‐specific knockout Alb‐Cre mice were C57BL/6J mice, all cKO mice used were in C57BL/6J background. Animals were maintained in a pathogen‐free facility under a 12/12 h light/dark cycle at 25 ± 1°C with a relative humidity of 50  ±  10% and anesthetized with an i.p. injection of 50 mg/kg BW of sodium pentobarbital.

### 4.2 Mouse TILI model

As previously reported,^22^ BALB/c mice were given daily oral doses of 80 mg/kg BW TSN. We prepared serum exosomal miRNAs according to the manufactures’ recommendations (see below) on days 3, 9, and 21 of TSN exposure.

### 4.3 Isolation and characterization of serum exosomes

We prepared exosomes from the serum of animals and cell culture experiments with the ExoQuick™ Exosome Precipitation Solution kit (System Biosciences, USA) according to the manufacturer's instructions. We determined the size of serum exosomes with the Zetasizer Nano S90 system (Malvern, UK) and imaged the morphology of exosomes with a JEM‐1230 transmission electron microscopy (TEM, JEOL, Japan) as previously reported.^46^ We evaluated markers of exosomes, that is, CD81, TSG101, and Syntenin by western blot analysis.

### 4.4 RNA extraction and microarray analysis of exosomal miRNAs

We isolated total RNA and miRNA with the Trizol reagent (Qiagen, Germany). We used the mirVana™ PARISTM Kit (Ambion, Inc., USA) to extract and purify exosomal miRNAs and measured whole genome expression levels of miRNAs with the Agilent mouse miRNA (8×60K) V21.0 array. The microarray data are available in Gene Expression Omnibus (GSE160961). We normalized the raw data with the GeneSpring GX 12.0 software (Agilent Technologies, USA) and applied the Student's *t*‐test to calculate statistical significance between the study groups. We selected the DEMs with the cutoff criteria *p *< 0.05 and a fold change >1.5 between the TSN treatment and control groups. Additionally, we performed a serum exosomal miRNA trajectory using PLS‐DA.

### 4.5 RNA extraction and RT‐qPCR

#### 4.5.1| miRNA

We isolated total RNA along with miRNA with the Trizol reagent (Qiagen). miRNAs were reverse transcribed with the miScript II RT Kit (Qiagen) and we performed qPCR with the miScript SYBR Green PCR Kit (Qiagen) on an Eppendorf Mastercycler ep realplex (Eppendorf, Germany). The primers were obtained from Sango, China and the sequences are listed in Table S7. We quantified miRNAs by the 2^−ΔΔCt^ method with miR‐30e‐5p as a housekeeping gene. To determine the expression of miR‐106b‐5p in cells and exosomes, we employed the following protocol: we cultured 1.2 × 10^8^ BNL CL.2 or JS1 cells in 75 cm^2^ flasks for 24 h. Subsequently, we treated the BNL CL.2 and the JS1 cell cultures with 0.1 and 2 µM TSN, respectively for 48 h. Controls were treated with exosome‐depleted culture media. Forty‐eight hours later, we harvested the cells and extracted the exosomes from the supernatant for RNA extraction. For quantification of miR‐106b‐5p in exosomes, we used cel‐miR‐39‐3p as a comparator miRNA. To determination the expression of miR‐106b‐5p in cells, we used the internal control U6 as a normalizer. All assays were performed as duplicate measurements and experiments were independently repeated three times. HepaRG cells (1.2 × 10^8^) were cultured in 75 cm^2^ flasks for 24 h and then treated with 1 µM TSN for 24 h. The internal control U6 was used as a normalizer.

#### 4.5.2| mRNA

We used the Trizol reagent (Qiagen) to extract total RNA from cells and performed cDNA synthesis with the QuantiTect Reverse Transcription Kit (Qiagen) according to the manufacturer's guidance. We quantified gene‐specific transcripts (Table S7; Sango, China) in qPCR assay with the QuantiFast SYBR Green PCR Kit (Qiagen) and expressed the data relative to *Gapdh* by calculating the fold change with the 2^−ΔΔCt^ method.

### 4.6 Bioinformatic analysis

We used the IPA (USA) software to identify target genes of regulated miRNAs by applying the function “Target Filter” and used the “tox analysis” tool in IPA to define hepatotoxicity‐related target genes.

### 4.7 miR‐106b‐5p agomir and antagomir treatments of TSN and APAP‐injured mice

We purchased the agomir and antagomirs of miR‐106b‐5p and the NC from Guangzhou RiboBio Co., Ltd. (China). See Table S8 for sequence information. Notably, the NC has minimal sequence identity to the miR‐106b‐5p agomir and antagomir and enables an assessment of unspecific effects. We allocated BALB/c mice randomly into six groups of *n* = 5∼6 animals/group (TSN + agomir control group: *n* = 5; other groups: *n* = 6). Except for the controls, which received daily oral doses of 1% sodium carboxymethylcellulose, mice were treated with oral doses (gavage) of 80 mg TSN/kg BW for 16 days in addition to coadministration of miR‐106b‐5p agomir and antagomirs as follow (the treatment scheme diagram is shown in Figure S12): Controls received 400 µL saline i.v. injections every 3 days from day 1 onward (group 1). Animals exposed to TSN only received 400 µL saline i.v. injections every 3 days from day 1 onward (group 2). To account for the expression of miR‐106b‐5p in the resolution phase of TILI, and to assess the effects of miR‐106b‐5p on TILI, animals were treated with 10 nmol miR‐106b‐5p agomir i.v. injections every 3 days from day 4 onward (group 3). Vice versa, animals were treated with the agomir NC received 10 nmol NC i.v. injections every 3 days from day 4 onward (group 4). We also assessed the effects of the miR‐106b‐5p antagomir on TILI following i.v. injections of 100 nmol antagomir every 3 days from day 1 onward (group 5). Likewise, the antagomir NC received 100 nmol iv injections every 3 days from day 1 onward (group 6).

We allocated C57BL/6J mice randomly into four groups (APAP group: *n* = 3; other groups: *n* = 5). Mice in the control (group 1) and APAP (group 2) groups received 200 µL saline i.v. injection. Animals in the agomir NC (group 3) and the miR‐106b‐5p agomir (group 4) groups were treated with 10 nmol agomir NC or miR‐106b‐5p agomir i.v. injection, respectively. After 60 h, mice were administered once again with the above administrations and then fasted for 16 h. Subsequently, the animals were given a single i.p. injection of 350 mg/kg BW of APAP (groups 2, 3, and 4) or saline solution (group 1).

### 4.8 Cellular uptake of exosomes

We stained exosomes with the PKH67 Green Fluorescent Cell Linker Mini Kit (Sigma, USA) according to the manufacturer's recommendations. Briefly, exosomes were suspended in Diluent C followed by the addition of the PKH67 dye. BNL CL.2 cells were cultured in 96‐well plates at 5000 cells/well for 24 h. Subsequently, the cultures were treated with vehicle (control group) or 100 µg/mL of labeled exosomes for 0, 2, 6, 12, 24, and 48 h. We used the Hoechst 33342 dye (Beyotime, China) as a nuclear counter stain and imaged the cells with a fluorescence microscope (Olympus, Japan).

### 4.9 Luciferase assay

We obtained luciferase reporter plasmids from Guangzhou iGene Biotechnology Co., Ltd China, which contained the wild type or mutant 3′‐untranslated region of the *Vim* gene in the pEZX‐FR02 vector.

We cultured BNL CL.2 cells in 96‐well plates at a density of 1 × 10^4^ cells/well. After 24 h, we transfected each well with 250 ng plasmid and 50 nM siRNA and 0.25 µL Lipofectamine 2000 (Thermo Fisher Scientific, USA) dissolved in Opti‐MEM culture medium (Gibco, USA) for 6 h. After cells were cultured with Dulbecco's modified Eagle medium (DMEM) for 48 h, we assayed the activity of the plasmid with the Luc‐Pair™ Duo‐Luciferase Assay Kit (GeneCopoeia, USA) as follows: the cells were lysed, and lysates were transferred to a 96‐well black plate. First, we added 100 µL Luc substrate solution and incubated the plate at room temperature for 3 min and measured OD_Luc_ fluorescence on an Infinite M1000 Pro plate reader. After detection, we added 100 µL of the R‐luc substrate solution to each well and once again, we incubated the plate at room temperature for 3 min and measured the fluorescence intensity OD_R‐luc_ with a Infnite M1000 Pro plate reader. We calculated the relative fluorescence according to the formula according to the manufacturer's instructions: $\mathrm{Relative}\;\mathrm{luciferase}\;\mathrm{activity}\;=\frac{OD_{\mathrm{Luc}}}{OD_{R-\mathrm{luc}}}$.

### 4.10 Single cell preparation and scRNA‐seq analysis

We isolated hepatocytes from TSN and miR‐106b‐5p agomir treated mice with a two‐step collagenase perfusion technique. Briefly, the livers were perfused in situ with 37°C ethylene diamine tetraacetic acid solution, followed by sequential perfusion with a buffer solution containing 0.5 mg/µL collagenase IV (Sigma), 4% BSA, and 0.1 mg/mL DNase I (Roche, USA) for 5 min. Digested livers were passed through a 40 µm cell strainer and centrifuged at 50 g for 3 min. After removing red blood cells with the Red Blood Cell Lysis Solution (Miltenyi Biotec, German), the cells were resuspended with 40% percoll (Sigma) and centrifuged at 4°C and 150 g for 8 min. This step was repeated twice. Finally, we assessed cell viability using the trypan blue staining method.

The hepatocytes from three mice per group were pooled and loaded into the 10× Genomics Chromium Single Cell chips. Libraries were prepared using the Chromium Next GEM Single Cell 3′ Reagent Kits v3.1 (10× Genomics, USA) according to the manufacturer's instructions, and sequenced on a Novaseq6000 (Illumina, USA). The single‐cell RNA‐sequencing data have been deposited at Gene Expression Omnibus (GSE23147579). We used the CellRanger (Genomics, version 6.1.1) software and the CellRanger count module for map reads to align and quantify the 10× raw sequencing data and the mouse genome mm10 was served as the reference genome. We removed doublets by applying the doubletFinder_v3 function of the R (4.1.1) package DoubletsFinder (2.0.3)^47^ and considered cells expressing more than 200 and <4000 unique genes with less than 10% reads mapping to mitochondria. We removed data of erythroid cells that highly expressed Hbb‐bs and other hemoglobin genes and applied the Seurat package (4.0.4) in R for downstream data analyses, which included the principle component analysis by applying the RunPCA function. We generated the t‐SNE plot, violin plot, dot plot, and heatmap by RunTSNE, VlnPlot, DotPlot, and DoHeatmap in R, respectively.

### 4.11 GO enrichment analysis

Differentially expressed genes (DEGs) were selected with a cutoff of *p *< 0.05 and absolute logFC > 0.5 using the FindAllMarkers function for the single‐cell RNA sequencing data. We identified GO biological processes enriched terms for DEGs with clusterProfiler R package (4.0.5)^48^ and pvalueCutoff = 0.05.

### 4.12 siRNA gene silencing studies

We purchased *Vim* siRNA (sense, 5′‐GGAAUCCUUGCAGGAAGAA‐3′; antisense, 5′‐UUCUUCCUGCAAGGAUUCC‐3′) from Guangzhou RiboBio Co., Ltd. (China) and cultured BNL CL.2 cells in 96‐well plates at a density of 5 × 10^3^ cells/well for 24 h. We used 0.5 µL of the Lipofectamine 2000 (Thermo Fisher Scientific) reagent to transfect 2.5 pmol of *Vim* siRNA and followed the manufacturer's protocol.

### 4.13 Cell proliferation following *Vim* siRNA gene silencing

We cultured BNL CL.2 cells in 96‐well plates at 5 × 10^3^ cells/well. After seeding for 24 h, we transfected the cells with *Vim* siRNA for 48 h. Then, we treated the cell cultures with 0.1 µM TSN and in the case of controls with DMEM culture media. We determined the proportion of BrdU incorporation as described in the Supplementary Materials.

### 4.14 Whole‐body and liver‐specific *Vim* knockout mice

To induce TILI, *Vim* ^−/−^ and WT mice were orally administered TSN at 80 mg/kg BW once a day for 9 days. In the case of AILI, *Vim* ^−/−^ and WT mice were initially fasted for 16 h. Subsequently, the animals were given a single i.p. injection of 300 mg/kg BW of APAP. Likewise, we treated the *Vim*‐cKO and *Vim*‐Flox mice with a single APAP i.p. injection of 350 mg/kg BW. Because of differences in the genetic background, the APAP dose differed for the *Vim* ^−/−^ and WT mice (BALB/c background) and the *Vim*‐cKO and *Vim*‐Flox mice (C57BL/6J background).

### 4.15 Clinical DILI cases

We obtained permission from the Ethics Committee of Hannover Medical School (459‐09, 3381‐2016, 8368‐2019) and the Medical Faculty of Leipzig University (186‐30052011) to perform blood and tissue analysis.

We determined the expression of miR‐106b‐5p and its target gene *Vim* in two separate patient cohorts. First, we examined the expression of serum miR‐106b‐5p in suspected flupirtine cases of severe idiosyncratic liver injury. The analgesic flupirtine has been linked to cases of sFILI and details of the patient cohort and the outcome after NAC/prednisolone antidote treatment have been published (see Table S9 for patient characteristics^49^). Second, we performed immunohistochemistry to assess vimentin expression in liver biopsies of patients diagnosed with drug and/or herbal‐induced ALF requiring liver transplantation.

### 4.16 Statistical analysis

We used Graphpad Prism 7 (GraphPad Software, USA) to determine statistical significance. Data are shown as means ± standard deviation (SD) and the reported *p*‐values are based on two‐tailed unpaired Student's *t*‐test, two‐way ANOVA test, or one‐way ANOVA followed by Tukey's multiple‐comparisons test. A *p*‐value < 0.05 was considered significant.

Further information is given in the Supplementary Materials.

## AUTHOR CONTRIBUTIONS

*Conceptualization: equal; data curation: lead; formal analysis: lead; funding acquisition: equal; investigation: lead; methodology: equal; project administration: supporting; supervision: equal; writing—original draft: supporting; writing—review and editing: lead*: Xiaoyan Lu. *Data curation: supporting; investigation: supporting; methodology: equal; validation: lead; visualization: lead; writing—original draft: lead*: Lingqi Yu. *Methodology: supporting; validation: supporting*: Jie Zheng. *Methodology: supporting; visualization: supporting*: Anyao Li. *Methodology: supporting; validation: supporting*: Junying Li. *Methodology: supporting; validation: supporting*: He Lou. *Methodology: supporting; validation: supporting*: Wentao Zhang. *Methodology: supporting; validation: supporting*: Hui Guo. *Validation: supporting*: Yuzhen Wang. *Validation: supporting*: Xuemei Li. *Conceptualization: supporting; formal analysis: supporting; investigation: supporting; methodology, supervision: Equal*: Yue Gao. *Conceptualization: equal; formal analysis: supporting; funding acquisition: equal; investigation: supporting; project administration: lead; supervision: equal; writing—review and editing: supporting*: Xiaohui Fan. *Methodology: supporting; funding acquisition: supporting; project administration: supporting; supervision: equal; validation, writing—review and editing: supporting*: Jürgen Borlak. All authors have read and approved the final manuscript.

## CONFLICT OF INTEREST STATEMENT

The authors declare no conflict of interest.

## ETHICS STATEMENT

All animal experimental protocols were approved by the Animal Care and Use Committee of the Zhejiang University School of Medicine (approval number ZJU20210059). For clinic studies, we obtained permission from the Ethics Committee of Hannover Medical School (459‐09, 3381‐2016, 8368‐2019) and the Medical Faculty of Leipzig University (186‐30052011) to perform blood and tissue analysis. The written informed consent was obtained from all participants.

## Supporting information

Supporting Information

## Data Availability

The microarray data are available in Gene Expression Omnibus (GSE160961). The single‐cell RNA‐sequencing data have been deposited at Gene Expression Omnibus (GSE23147579). Any additional information required to reanalyze the data reported in this paper are available from the corresponding authors.
