# Supplementary material for: miR‐106b‐5p protects against drug‐induced liver injury by targeting vimentin to stimulate liver regeneration
Source: MedComm (2020). 2024 Aug 21;5(9):e692. doi: 10.1002/mco2.692 (PMC11337467; doi:10.1002/mco2.692)
Supplement: Supplementary file 1 — Supporting Information [file MCO2-5-e692-s001.docx]

Supplemental information

miR-106b-5p protects against drug-induced liver injury by targeting vimentin to stimulate liver regeneration

Xiaoyan Lu^1,2,3,#^, Lingqi Yu^1,2,#^, Jie Zheng^1^, Anyao Li^1^, Junying Li^1^, He Lou^1^,

Wentao Zhang^4^, Hui Guo^4^, Yuzhen Wang^5^, Xuemei Li^3^, Yue Gao^3,6,*^,

Xiaohui Fan^1,2,3,7,*^, and Jürgen Borlak^8,*^

Affiliations:

^1^Pharmaceutical Informatics Institute, College of Pharmaceutical Sciences, Zhejiang University, Hangzhou, 310058, China;

^2^State Key Laboratory of Chinese Medicine Modernization, Innovation Center of Yangtze River Delta, Zhejiang University, Jiaxing, 314100, China;

^3^State Key Laboratory of Component-Based Chinese Medicine, Tianjin University of Traditional Chinese Medicine, Tianjin, 301617, China;

^4^Department of Hepatobiliary, the First Affiliated Hospital of Tianjin University of Traditional Chinese Medicine, Tianjin, 300193, China;

^5^Department of Pharmacy, Sir Run Run Shaw Hospital, Zhejiang University School of Medicine, Hangzhou, 310016, China;

^6^Department of Pharmaceutical Sciences, Beijing Institute of Radiation Medicine, Beijing, 100850, China;

^7^The Joint-laboratory of clinical multi-omics research between Zhejiang University and Ningbo Municipal Hospital of TCM, Ningbo Municipal Hospital of TCM, Ningbo, 315012, China;

^8^Centre for Pharmacology and Toxicology, Hannover Medical School, Carl-Neuberg-Str. 1, 30625, Hannover, Germany.

^*^Corresponding authors

E-mail addresses: [gaoyue@bmi.ac.cn](mailto:gaoyue@bmi.ac.cn) (Yue Gao), [fanxh@zju.edu.cn](mailto:fanxh@zju.edu.cn) (Xiaohui Fan), [Borlak.Juergen@mh-hannover.de](mailto:Borlak.Juergen@mh-hannover.de) (Jürgen Borlak)

^#^The authors contributed equally to this work.

**Materials and Methods**

## Serum biochemistry

We assayed serum alanine aminotransferase (ALT) and aspartate aminotransferase (AST) activities with the Colorimetric Assay Kit (Biovision, USA) according to the manufacturer’s instructions.

## Cell culture

We obtained the mouse BNL CL.2 hepatocytes and human HepG2 cells from the Cell Bank of Shanghai Institutes for Biological Sciences, Chinese Academy of Sciences. Human HepaRG cells were purchased from the Shanghai BinSui Biological Technology Co., Ltd. (Shanghai, China). JS1 hepatic stellate cells were the gift from Jinsheng Guo, Shanghai Medical College, Fudan University, China.^1^ We cultured cells (P5-P15) in Dulbecco’s Modifed Eagle Medium (DMEM, Gibco, USA) supplemented with 10% fetal bovine serum (FBS, Gibco, USA) and a 1% penicillin-streptomycin mixture (100×Penicillin-Streptomycin Solution, Gibco, USA).

## 5-Bromo-20-deoxyuridine (BrdU) assay

We cultured BNL CL.2 cells in 96-well plates at 5×10^3^ cells/well. Twenty-four hours after seeding, we treated the cells with TSN and miR-106b-5p as follows:

1. Control group: received culture media only

2. TSN model group: 0.1µM TSN

3. Antagomir control group: 0.1µM TSN and 100 nM negative control (NC)

4. Antagomir miR-106b-5p treatment group: 0.1µM TSN and 100 nM antagomir miR-106b-5p

5. Agomir control group: 0.1µM TSN and 100 nM NC

6. Agomir miR-106b-5p treatment group: 0.1µM TSN and 100 nM agomir miR-106b-5p.

Notably, NCs have minimal sequence identity to the miR-106b-5p agomir and antagomir and enable an assessment of unspecific effects.

Each group consisted of five repetitive wells. After TSN administration, cells were incubated for 48 h, and we determined the proliferation with the BrdU assay (Roche, USA) according to the manufacturer’s instructions. In brief, the cells were labelled with BrdU followed by an incubation step with the FixDenat solution and the Anti-BrdU POD working solution. Thereafter, we removed the working solution, washed the cells repeatedly with PBS and added 100 µL of peroxidase substrate solution for an incubation period of 10 min. Finally, we monitored the incorporation of BrdU into DNA and measured the absorbance at 492 nm and 370 nm using an Infnite M1000 Pro (TECAN, Germany) plate reader. We calculated the proportion of BrdU incorporation as follows:

$$\text{ΔOD=OD(370 nm)-OD(492 nm)}$$

$$\text{BrdU incorporation(\%)=}\frac{\text{ΔOD}_{\text{treatment}}\text{-}\text{ΔOD}_{\text{blank}}}{\text{Δ}\text{OD}_{\text{control}}\text{-}\text{ΔOD}_{\text{blank}}}\text{×100}$$

## Annexin V/PI double fluorescence staining

We cultured BNL CL.2 cells in 6-well plates at a density of 1.5×10^5^ cells/well for 24 h. Subsequently we treated the cell cultures as follows:

1. Control group: received culture media only

2. TSN model group: 0.1 µM TSN

3. Antagomir control group: 0.1µM TSN and 100 nM NC

4. Antagomir miR-106b-5p treatment group: 0.1µM TSN and 100 nM antagomir miR-106b-5p

5. Agomir control group: 0.1µM TSN and 100 nM NC

6. Agomir miR-106b-5p treatment group: 0.1µM TSN and 100 nM agomir miR-106b-5p.

We used the FITC Annexin V Apoptosis Detection Kit I (BectonDickinson, USA) to determine apoptotic cells with PI as a nuclear counterstain according to the manufacturer’s protocol. Briefly, cells were digested and resuspended in 1 × Binding Buffer solution. Next, we added 5 µL Annexin V-FITC and 5 µL PI dye and allowed for an incubation period of 15 min at room temperature. Finally, we quantified the stained cells with a BD Accuri™ C6 Plus Flow Cytometer (BD, USA).

## Cell survival rate

We cultured HepG2 cells in 96-well plates at a density of 5×10^3^ cells/well for 24 h. Subsequently we treated the cell cultures as follows:

1. Control group and triptolide model group: received culture media only

2. Agomir control group: 400 nM NC

3. Agomir miR-106b-5p treatment group: 400 nM agomir miR-106b-5p.

After 6 h of incubation, the triptolide model group, agomir control group, and agomir miR-106b-5p treatment group were added 100 nM triptolide. The survival rate of HepG2 cells was examined using the CCK-8 assay after 24 h.

## Cell proliferation following *Vim* siRNA gene silencing

We cultured BNL CL.2 cells in 96-well plates at 5×10^3^ cells/well. After seeding for 24 h, we transfected the cells with *Vim* siRNA for 48 h. Subsequently, we treated the cell cultures with 0.1 µM TSN and in the case of controls with DMEM culture media. We determined the proportion of BrdU incorporation as described above.

## Western blot

BNL CL.2 cells were washed 3 times with PBS and lysed in Western and IP lysis buffer containing a protease inhibitor cocktail (Roche Diagnostics, Germany). Livers of APAP-injured mice were lysed in RIPA lysis buffer (Beyotime, China). We determined the protein concentration with the Bicinchoninic Acid Protein Assay Kit (Thermo Fisher Scientific, USA) and performed sodium dodecyl sulfate-polyacrylamide gel electrophoresis (SDS-PAGE) on a 10% gel. Following SDS-PAGE, the samples were transferred to a polyvinyliden difluoride membrane and after blocking with TBST (Tris-buffered saline, 0.1% Tween 20) containing 5% non-fat dry milk. We incubated the membranes with the primary antibody against vimentin (Cell Signaling Technology Cat# 5741S, RRID: AB_10695459), and CYP2E1 (Proteintech Cat# 19937-1-AP, RRID: AB_10646444). Following an incubation step with horseradish-peroxidase (HRP)-conjugated antibody for 1 h at room temperature, we applied an enhanced chemiluminescent substrate reagent (Thermo Fisher Scientific, USA) and the protein bands were visualized and digitized with a charge-coupled device camera (Bio-Rad, USA).

## Histological, immunohistochemical and immunofluorescence analyses

To assess injury in the liver, paraffin-embedded liver sections were stained with hematoxylin and eosin (H&E). We assessed DNA fragmentation in liver sections by the terminal deoxynucleotidyl transferase-mediated dUTP-biotin nick end labeling (TUNEL) assay using an *In Situ* Cell Death Detection Kit (Roche Diagnostics, USA) according to the manufacturer’s instructions and we determined cell proliferation with an anti-Ki67 antibody (Abcam Cat# ab16667, RRID: AB_302459; Cell Signaling Technology Cat# 12202T, RRID: AB_2620142) according to the manufacturer’s guidance. We used an anti-vimentin antibody (Abcam Cat# ab92547, RRID: AB_10562134), an anti-cytokeratin 18 antibodys (Abcam Cat# ab668, RRID: AB_305647; Proteintech Cat# 10830-1-AP, RRID: AB_2133164), and an anti-Thymosin beta 4 antibody (Proteintech Cat# 19850-1-AP) to determine their expression by immunofluorescence in liver sections using standard operating procedures.


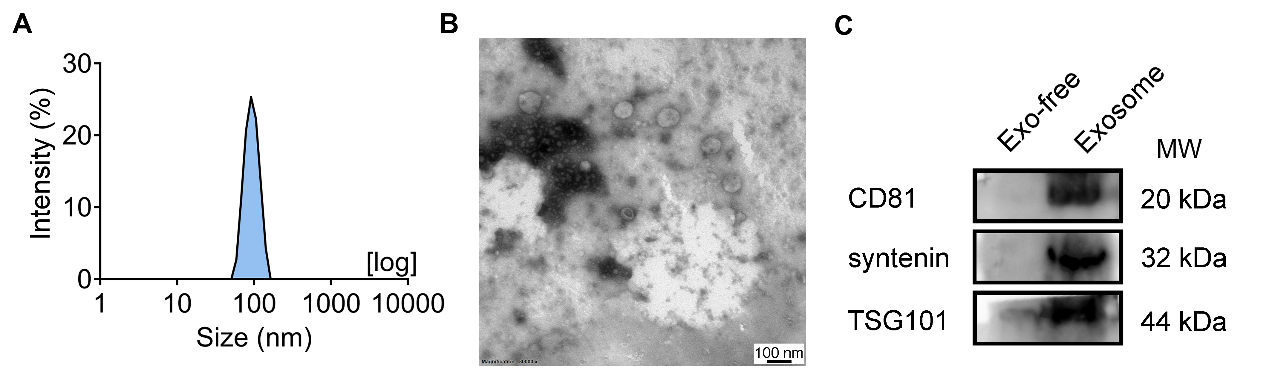


**Figure S1** Characterization of serum exosomes from mice. (A) Shown is the size distribution of serum-derived exosomes. (B) Shown is a representative TEM image of isolated exosomes. The scale bar is 100 nm. (C) WB experiments confirm the expression of the CD81, syntenin, and TSG101 in serum-derived exosomes. The exosome markers are not expressed in exosome free serum.


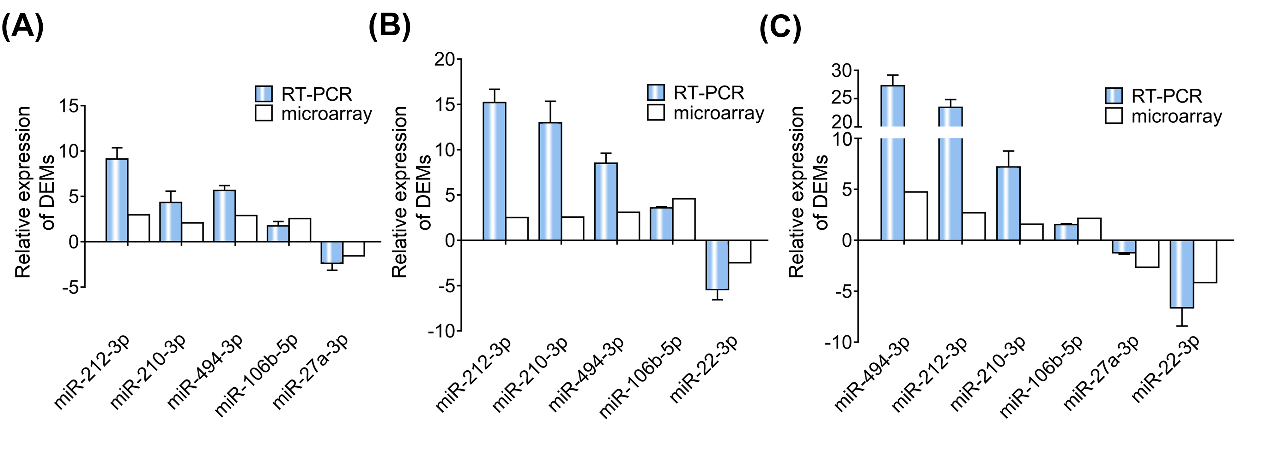


**Figure S2** Validation of the differentially expressed miRNAs (DEMs) by real-time qPCR. (A) Regulated miRNAs at day 3 of TSN treatment were selected at random. There is good agreement between the two platforms. (B) Regulated miRNAs at day 9 of TSN treatment were selected at random. There is good agreement between the two platforms. (C) Regulated miRNAs at day 21 of TSN treatment were selected at random. There is good agreement between the two platforms. Blue bars indicate the mean fold change (± SD) from 3 independent real-time quantitative PCR experiments. White bars indicate the microarray data.


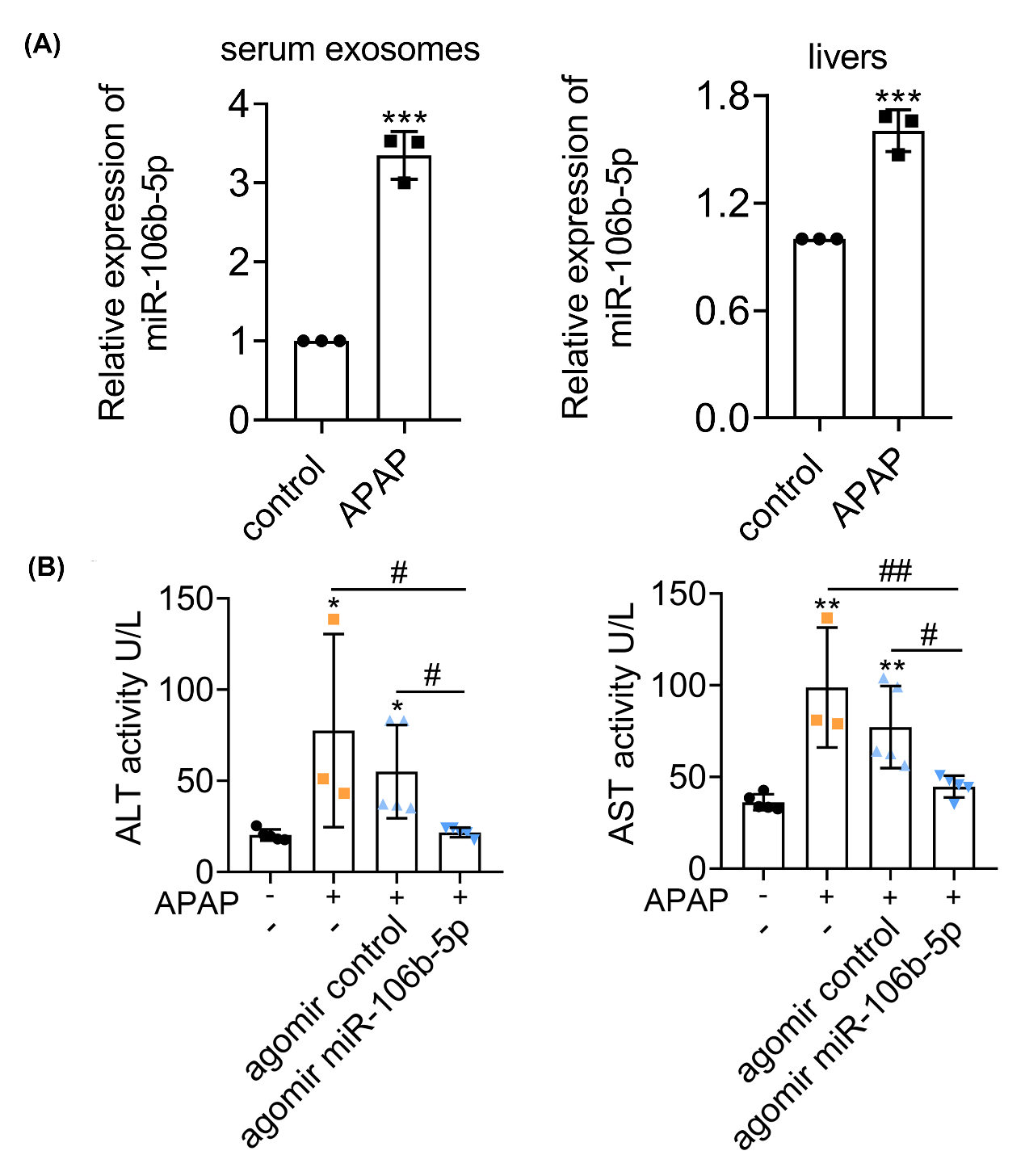


**Figure S3** miR-106b-5p could protect mice from APAP-induced liver injury. (A) The expressions of miR-106b-5p in serum exosomes (left panel) and livers (right panel) of APAP-induced liver injury mice. (B) Serum ALT and AST activities in APAP-injured mice. Control group, APAP + agomir control group, APAP + agomir miR-106b-5p group, n = 5. APAP group, n = 3. The significance was determined with an unpaired Student's t-test. Compared with the control group, ^*^ *P* < 0.05, ^**^ *P* < 0.01, ^***^ *P* < 0.001. The composition of the two groups marked with horizontal lines, ^#^ *P* < 0.05, ^##^ *P* < 0.01.


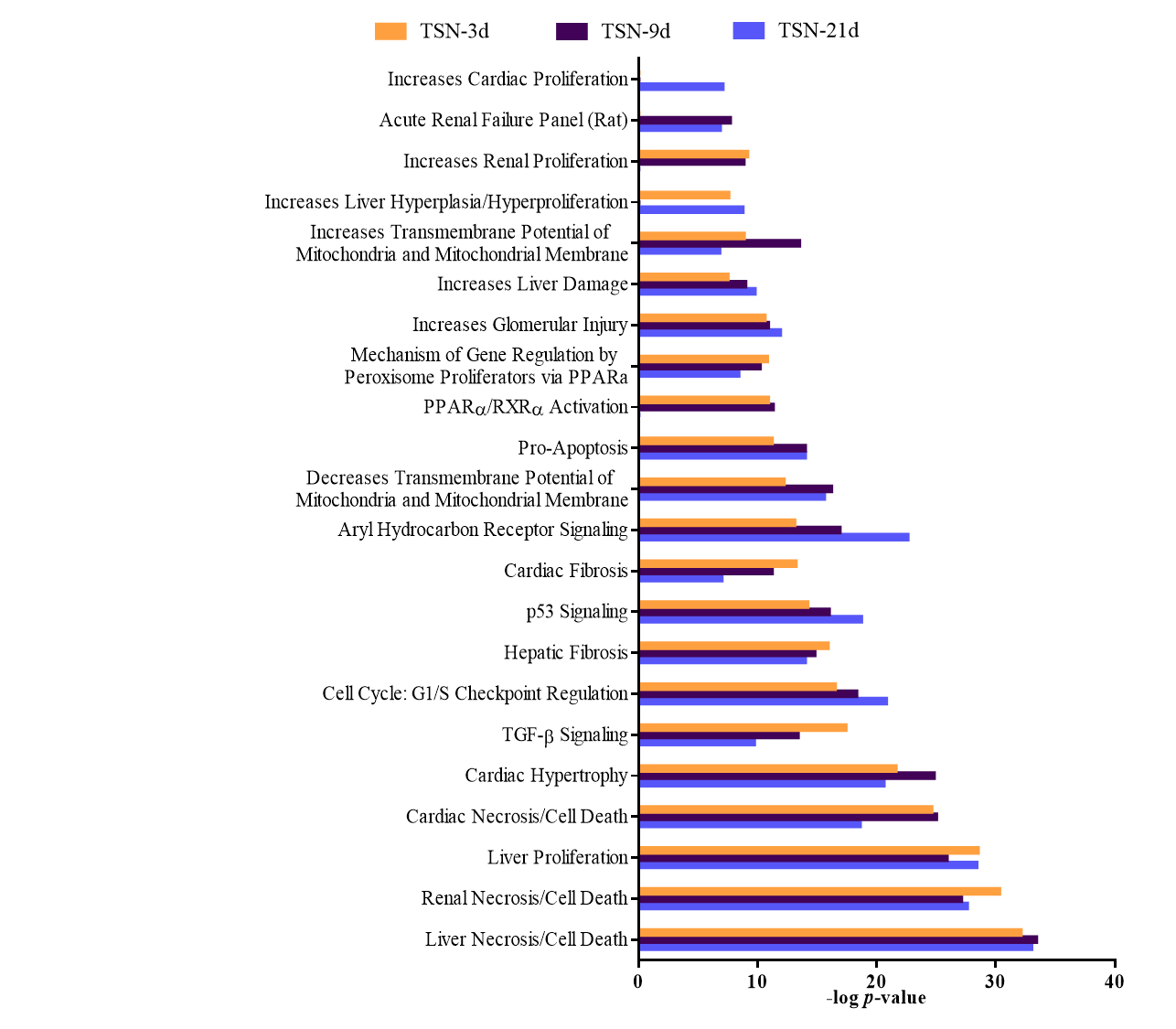


**Figure S4** Toxic lists enriched for target genes of serum exosomal DEMs after 3, 9, and 21 days of TSN administration.


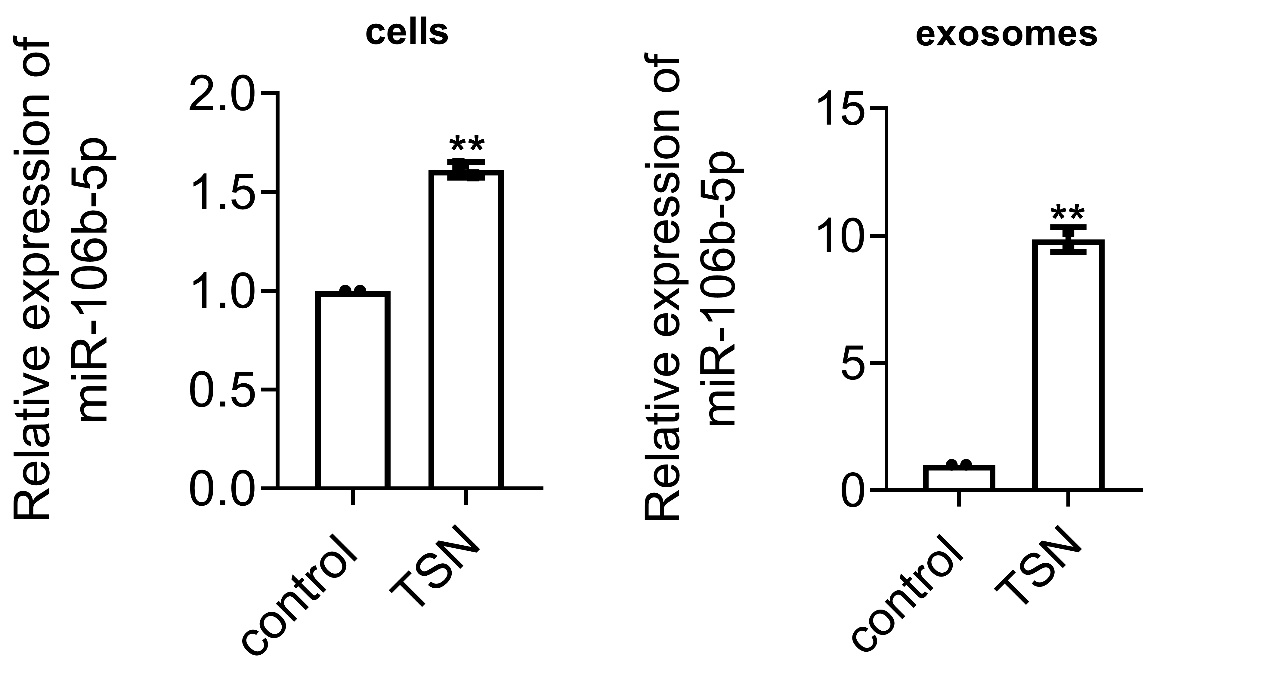


**Figure S5** The expressions of miR-106b-5p in cells (left panel) and supernatant exosomes (right panel) of HepaRG after TSN exposure. The significance was determined using an unpaired Student's t-test. Compared with the control group, ^**^ *P* < 0.01.


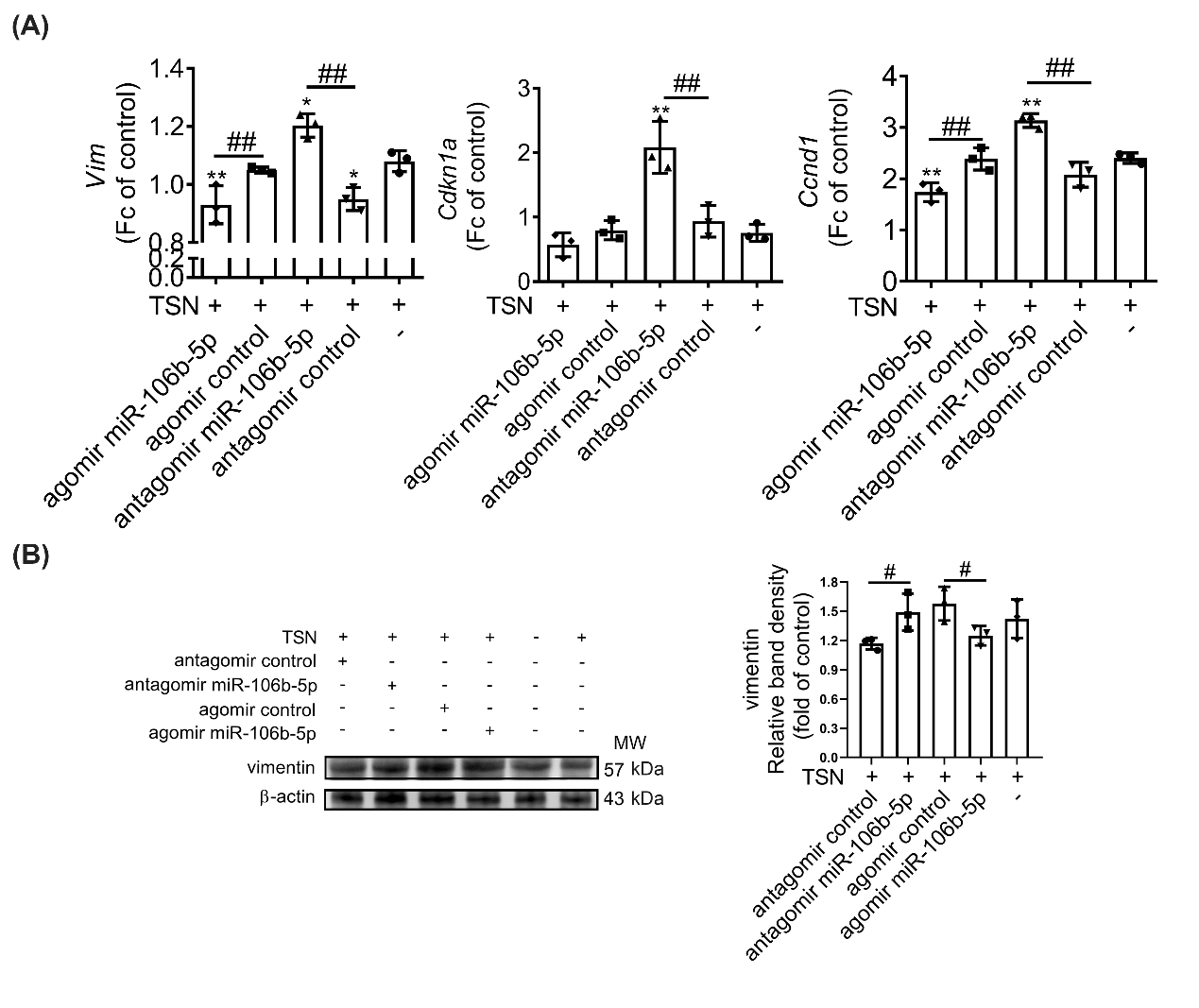


**Figure S6** miR-106b-5p decreases mRNA and protein expressions of its target genes. (A) The mRNA expression levels of *Vim*, *Cdkn1a*, and *Ccnd1* in liver sections of mice on day 16. (B) Vimentin expression in liver sections of mice on day 16 measured using western blot. The significance was determined with a one-way ANOVA followed by Tukey’s multiple-comparison test (A) or two-tailed unpaired Student’s t-test (B). Compared with the TSN group, ^*^ *P* < 0.05, ^**^ *P* < 0.01. The composition of the two groups marked with horizontal lines, ^#^ *P* < 0.05, ^##^ *P* < 0.01.


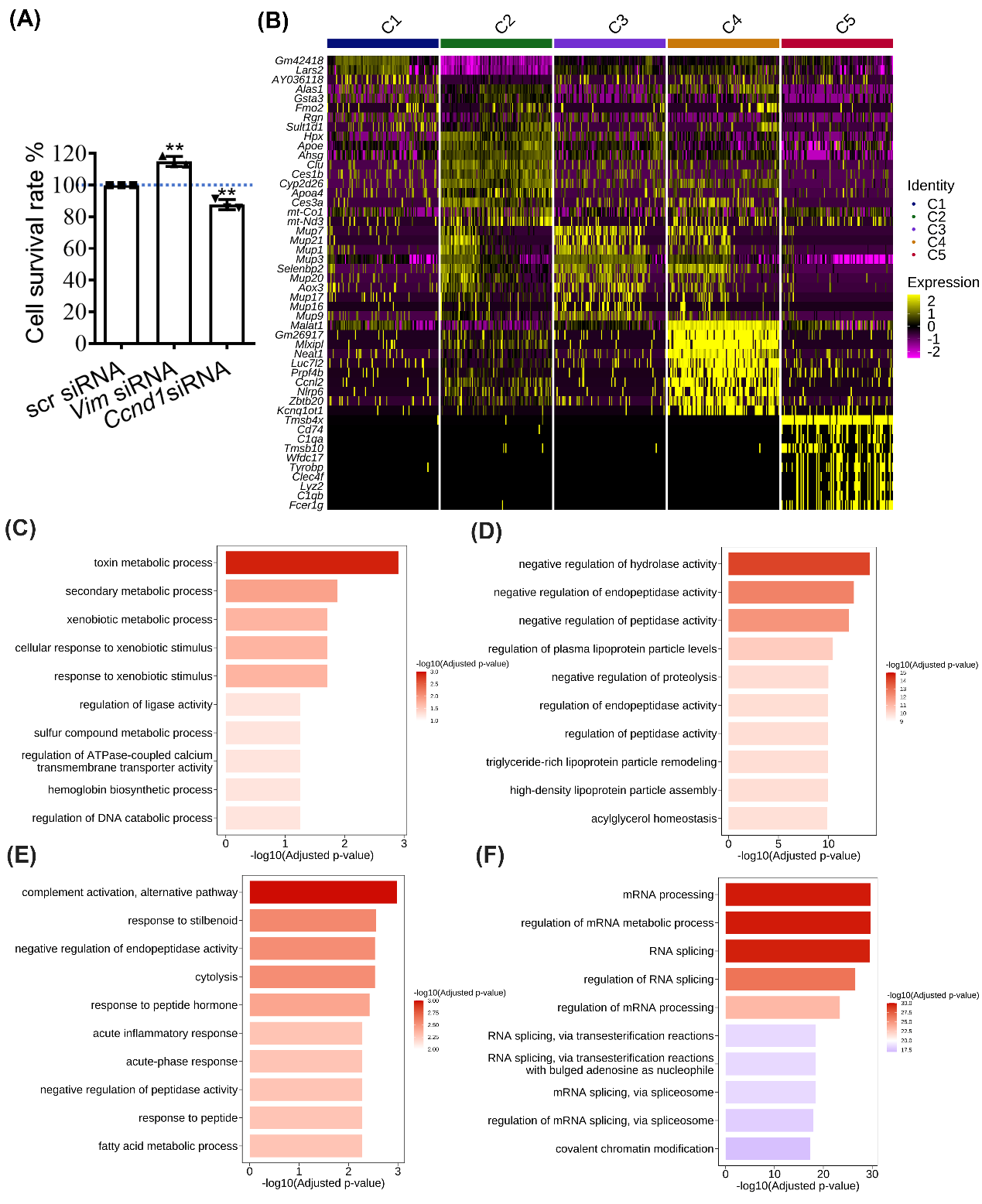


**Figure S7** *Vim* is a direct target of miR-106b-5p. (A) We determined the cell viability with the MTT and the data are means ± SD (n = 3). We determined statistical significance with a one-way ANOVA followed by Tukey’s multiple-comparison test. Compared with the scr siRNA group,^**^ *P* < 0.01. (B) Heatmap of top differentially expressed genes in each hepatocyte subset determined by single-cell RNA sequencing. (C) GO enrichment terms of C1. (D) GO enrichment terms of C2. (E) GO enrichment terms of C3. (F) GO enrichment terms of C4.


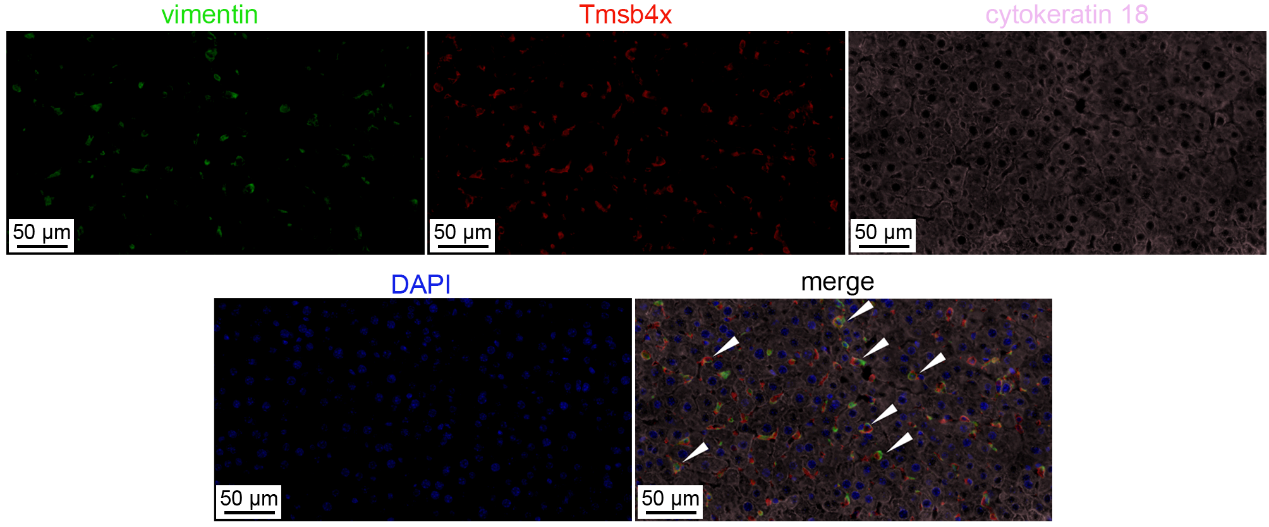


**Figure S8** Immunofluorescent staining of vimentin, Tmsb4x, and cytokeratin 18 in the liver sections of the mice treated with TSN. Cells simultaneously stained with green, blue, and pink fluorescence with arrows are C5 hepatocytes expressing vimentin. The scale bar is 50 μm.


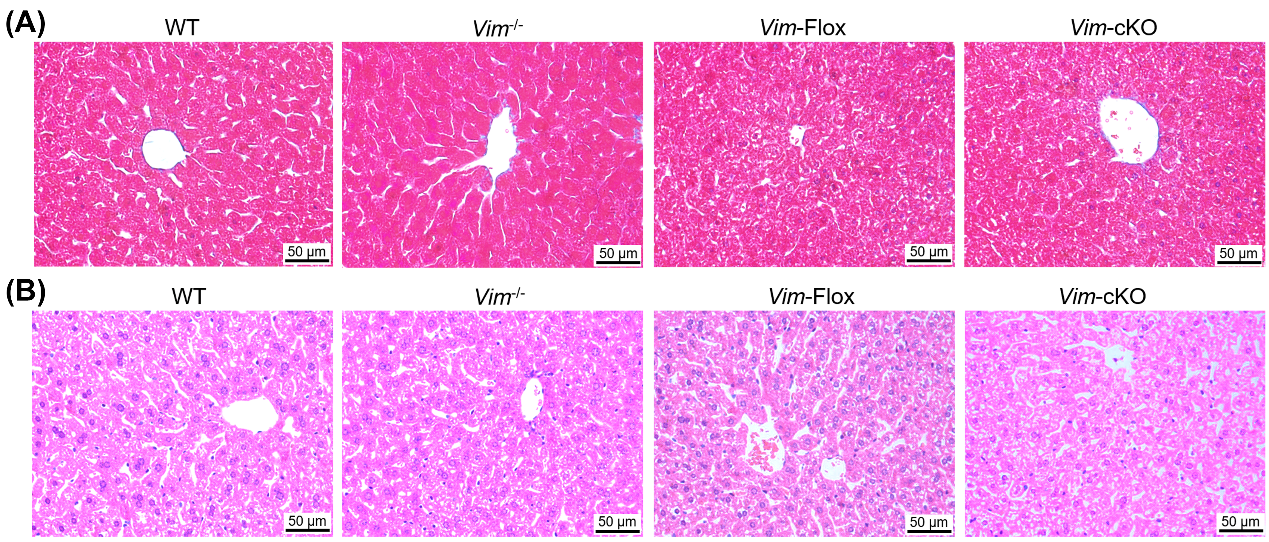


**Figure S9** Lacking vimentin has no obvious influence on the reproduction and development of mice. (A) Masson staining of *Vim*^-/-^ or liver-specific *Vim*-cKO mice. (B) H&E staining of *Vim*^-/-^ or liver-specific *Vim*-cKO mice. Magnification: 400×. The scale bar is 50 μm.


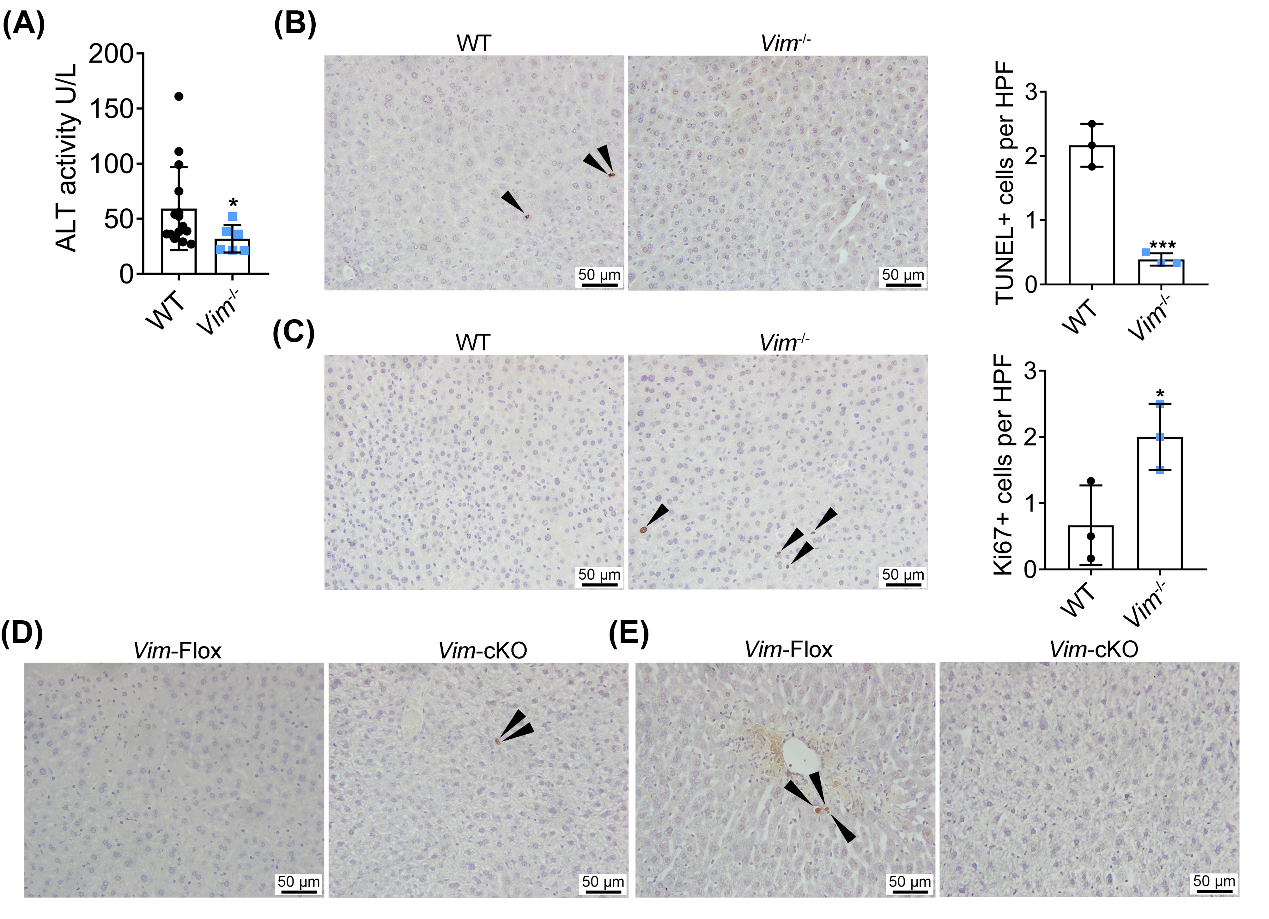


Figure S10 *Vim* knockout mice are protected from TILI and AILI. TSN treatment (TILI): *Vim* knockout mice were treated daily by intragastric dosing of 80 mg/kg TSN for 9 days. Acetaminophen treatment (AILI): *Vim* knockout mice were treated with a single ip dose of 300 mg/kg acetaminophen and killed after 24 h. (A) Serum ALT activity in mice treated with TSN. WT group, n = 15. *Vim*^-/-^ group, n = 6. We tested statistical significance with an unpaired Mann-Whitney *U* test and compared the data to WT mice. (B) TUNEL staining of liver sections in TSN treated mice. Left panel: Representative images (400×); brown cells with arrows are TUNEL positive. Right panel: Proportion of brown positive cells. We tested statistical significance with a two-tailed unpaired Student’s t-test and compared the data to WT mice. (C) Ki-67 staining of liver sections of TSN treated mice. Left panel: Representative images (400×); brown cells with arrows are Ki-67 positive. Right panel: Proportion of Ki-67 positive cells. We tested statistical significance with a two-tailed unpaired Student’s t-test and compared the data to WT mice. (D) Ki-67 immunohistochemical staining of liver sections of acetaminophen treated mice. (E) TUNEL staining of liver sections of acetaminophen treated. ^*^*P* < 0.05, ^***^*P* < 0.001.


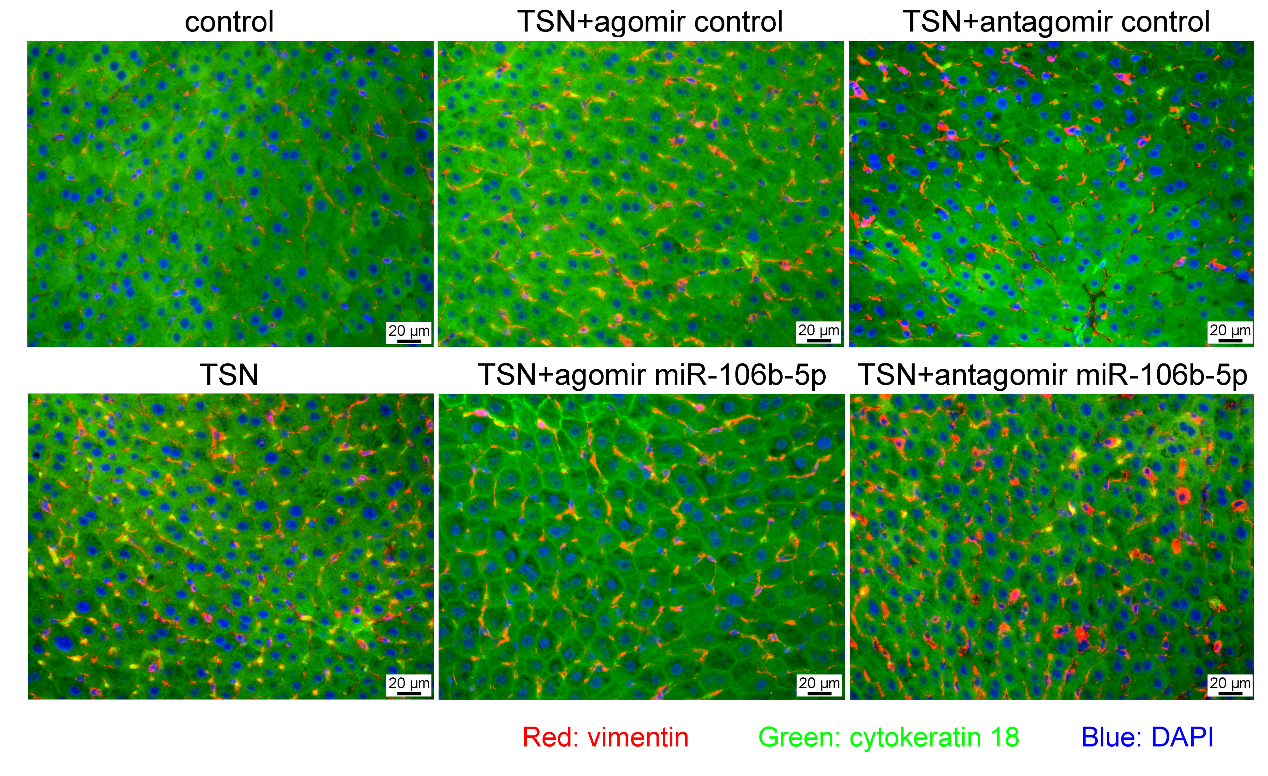


Figure S11 Vimentin is expressed in the hepatocytes of TILI mice. Immunofluorescent staining of vimentin in the liver sections of the mice co-treated with TSN and agomir or antagomir miR-106b-5p on day 16.

**
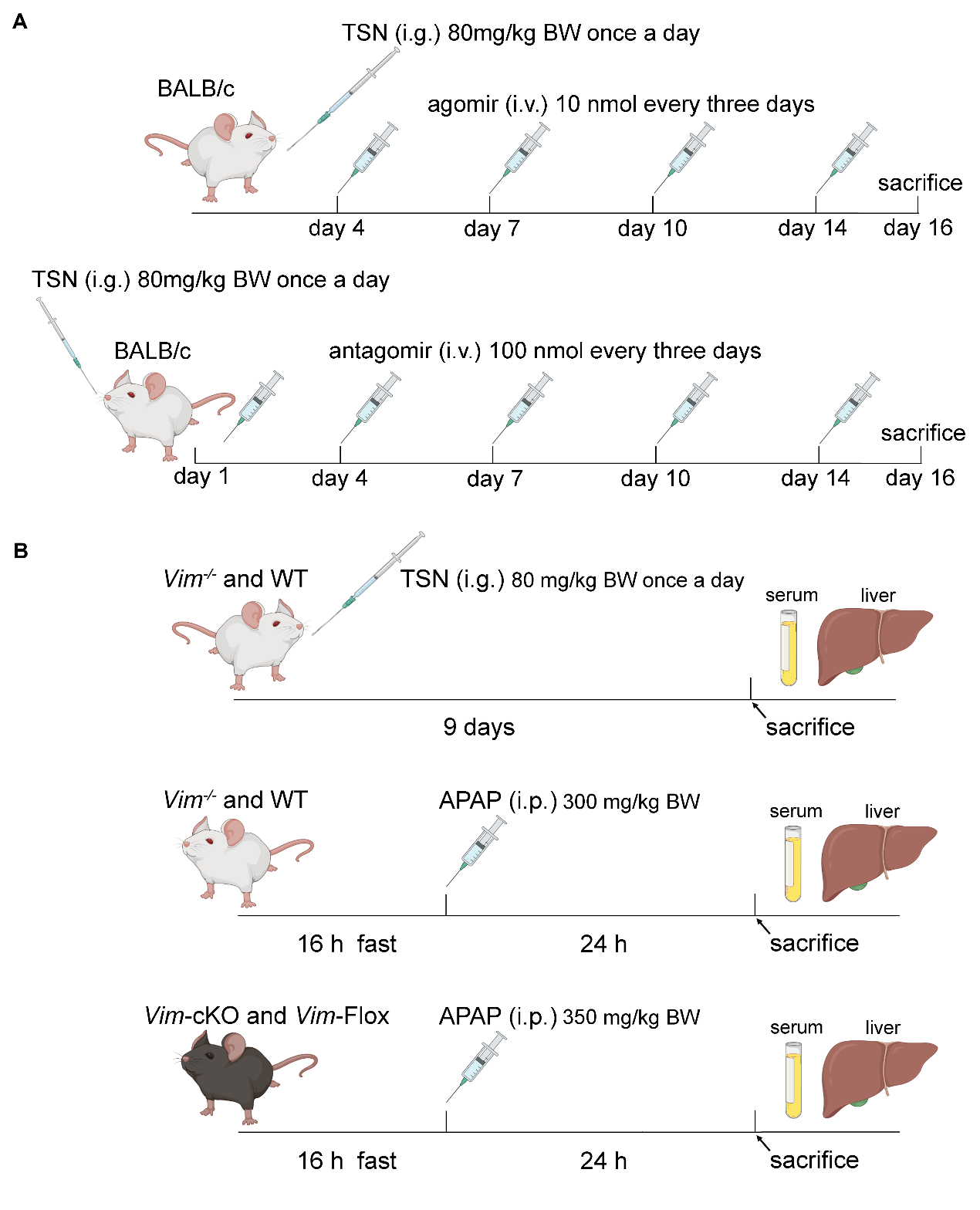
**

**Figure S12** Treatment scheme. (A) miR-106b-5p agomir and antagomir treatments of TSN injured mice. (B) Liver-specific and whole-body *Vim* knockout mice treatment schedules.

**Table S1. Fold change of ALT activity^#^**

| **Days** | **Mean ± SEM** | **95% CI** |
| --- | --- | --- |
| day 3 | 1.464 ± 0.1287 | 1.196 to 1.733 |
| day 9 | 4.175 ± 1.204 | 1.678 to 6.672 |
| day 21 | 1.465 ± 0.1493 | 1.155 to 1.774 |

**^#^**Control group, n = 21 (day 3 and day 9), n = 20 (day 21). TSN group, n = 21 (day 3), n = 23 (day 9 and day 21). Part of the data is from our previously published paper.^2^

**Table S2. Fold change of AST activity^#^**

| **Days** | **Mean ± SEM** | **95% CI** |
| --- | --- | --- |
| day 3 | 1.769 ± 0.1095 | 1.541 to 1.998 |
| day 9 | 8.278 ± 1.726 | 4.697 to 11.86 |
| day 21 | 1.624 ± 0.2102 | 1.188 to 2.060 |

**^#^**Control group, n = 21 (day 3 and day 9), n = 20 (day 21). TSN group, n = 21 (day 3), n = 23 (day 9 and day 21). Part of the data is from our previously published paper.^2^

**Table S3 DEMs that contribute to the top 20 toxic lists**

| **Days** | **miRNAs** |
| --- | --- |
| day 3 | let-7i-5p, miR-106b-5p |
| day 9 | let-7d-5p, miR-21a-5p, miR-106b-5p |
| day 21 | miR-21a-5p, miR-106b-5p |

**Table S4 The expressions of miR-17 family on 3, 9, and 21-day TSN exposure**

| **miRNAs** | **day 3** | **day 9** | **day 21** |
| --- | --- | --- | --- |
| miR-106b-5p | 2.56 | 4.58 | 2.16 |
| miR-17-5p | / | 2.82 | / |
| miR-20a | / | / | / |
| miR-93-5p | / | 2.41 | / |
| miR-20b | / | / | / |
| miR-106a | / | / | / |

“/” was used to indicate that the expression of miRNA did not change significantly (absolute fold change < 2).

**Table S5. Forty-four validated targets of miR-106b-5p in IPA**

| **Validated target genes of miR-106b-5p in IPA** | | |
| --- | --- | --- |
| *Vim* | *Bnip2* | *Pak7* |
| *Ccnd1* | *Creb1* | *Pkd2* |
| *Tlr7* | *Cxcel8* | *Pparg* |
| *Cdkn1a* | *E2f1* | *Pten* |
| *Mylip* | *E2f2* | *Pura* |
| *App* | *Esr1* | *Rb1* |
| *Bcl2l11* | *Vegfa* | *Rbl2* |
| *Bcl2* | *Hbp1* | *S1pr1* |
| *Crim1* | *Hipk3* | *Stat3* |
| *Runx1* | *Itch* | *Tgfb2* |
| *Jak1* | *Map3k12* | *Tnf* |
| *E2f3* | *Mef2d* | *Tp63* |
| *Arid4b* | *Mica* | *Tusc2* |
| *BambI* | *Mmp3* | *Zbtb7a* |
| *Bmpr2* | *Ncoa3* |  |

**Table S6. Patient demographics of acute liver failure cases due to DILI.**

| **Patient history** | | | | | | |  | **Serum biochemistries at admission** | | | | | | | **Histopathological information and immunostaining results of vimentin** |
| --- | --- | --- | --- | --- | --- | --- | --- | --- | --- | --- | --- | --- | --- | --- | --- |
|  | **Sex**  **[f/m]** | **Age** | **BMI** | **Suspected reason for ALF-LT** | **Running medications** | **Symptomes before admission [days]** | **Encephalopathia at admission** | **AST (U/l)** | **ALT (U/l)** | **Bilirubin (µmol/l)** | **Urea (mmol/l)** | **Lactate (mg/dl)** | **Quick (%)** | **NH3 (mmol/l)** |  |
| Fig. 7 panel Bi | m | 51-60 | 20.3 | DILI | Phyto-medication (celandine) | 28 | yes | 818 | 1136 | 275 | 5.8 | 1.97 | 26 | 71 | A case of celandine (Chelidonium majus) suspected herbal liver injury. This is a 51-60 year old male liver failure case characterized by bridging fibrosis and proliferation of neocholangioles with conversion of hepatocytes into ductal biliary epithelial cells (=metaplasia) to indicate bile duct regeneration. The fibrous septa formation marks subacute hepatic dystrophy with loss of the trabecular framework, extensive parenchymal destruction, necrosis with ball-like bilayered hepatocytes undergoing degeneration. The Kupffer cells are activated with marked expression of vimentin. At high power filed magnification hepatic stellate cells appear activated (= myofibroblast like) with strong expression of vimentin. |
| Fig. 7 panel Bii | m | 31-40 | 21.6 | DILI | Ibuprofen, Iberogast, Pantoprazol, Cannabis | 22 | yes | 1119 | 1412 | 688 | 1.7 | 2.8 | 20 | \ | A case of a 31-40 year old male diagnosed with severe liver injury putatively linked to chronic daily cannabis consumption. Marked inflammatory cell infiltrates and focal aggregates are seen indicating the development of hepatic granulomas. Inflammatory cells and harmed hepatocytes express vimentin. |
| Fig. 7 panel Biii | f | 41-50 | 26.9 | DILI | Diclofenac, Ibuprofen | 7 | no | 3627 | 3824 | 318 | 3.7 | 2.5 | 37 | 61 | A case of diclofenac suspected drug-induced liver injury. This is a 41-50 year old female diagnosed with acute liver failure and severe hepatic dystrophy. Shrunken hepatocytes and activated Kupffer cells express vimentin. A distinct vimentin expression just underneath the plasma membrane of inflammatory cell infiltrates is seen. The bile ducts are intact with destroyed hepatocytes around the portal field. |
| Fig. 7 panel Biv | f | 41-50 | 26.1 | DILI | Kava-kava, sometimes Paracetamol | 7 | yes | 769 | 1087 | 388 | 2.3 | 7.82 | 8 | 63 | A case of kava kava suspected herbal liver injury. This is a 41-50 year old female diagnosed with severe hepatic dystrophy, proliferative and intact bile ducts; destroyed hepatocytes around portal fields. Acute hepatitis is hallmarked by mixed vimentin positive inflammatory cell infiltrates. |
| Fig. 7 panel Bv | f | 41-50 | 33.1 | DILI | Phenprocoumon, Thyroxin, Ranitidin | 3 | no | 1844 | 1987 | 115 | 2.8 | 1.27 | 4 | \ | A case of phenprocoumon (=4-hydroxycoumarin) suspected liver injury. This is a 41-50 year old female diagnosed with acute liver failure, parenchymal necrosis, mixed inflammatory infiltrates of vimentin positive lymphocytes and neutrophils, necro-inflammatory foci and proliferative fibrous septa formation. |

**Table S7. Primer sequences**

|  | **Name** | **5’-3’** |
| --- | --- | --- |
| **miRNAs** | miR-212-3p | TAACAGTCTCCAGTCACGGCCA |
|  | miR-210-3p | CTGTGCGTGTGACAGCGGCTGA |
|  | miR-494-3p | TGAAACATACACGGGAAACCTC |
|  | miR-106b-5p | TAAAGTGCTGACAGTGCAGAT |
|  | miR-27a-3p | TTCACAGTGGCTAAGTTCCGC |
|  | miR-22-3p | AAGCTGCCAGTTGAAGAACTGT |
|  | miR-30e-5p | TGTAAACATCCTTGACTGGAAG |
|  | U6 | CTCGCTTCGGCAGCACA |
|  | cel-miR-39-3p | TCACCGGGTGTAAATCAGCTTG |
| **Genes** | *Bcl2l11* | Forward: GCCAGGCCTTCAACCACTAT  Reverse: TGCAAACACCCTCCTTGTGT |
|  | *Vim* | Forward: CCTGAGAGAAACTAACCTGGAGTC  Reverse: GCACTGTTGCACCAAGTGTGT |
|  | *Gapdh* | Forward: GTCAAGCTCATTTCCTGGTATG  Reverse: GGATAGGGCCTCTCTTGCTC |

**Table S8. The sequences of agomir and antagomir miR-106b-5p**

| **5’-3’** | | |
| --- | --- | --- |
| agomir miR-106b-5p | Forward: UAAAGUGCUGACAGUGCAGAU  Reverse: AUCUGCACUGUCAGCACUUUA | |
| antagomir miR-106b-5p | | AUCUGCACUGUCAGCACUUUA |

**Table S9.** **Patient demographics of severe idiosyncratic liver injury case**

| **Patient ID** | **Age [yrs]** | **Sex**  **[f/m]** | **BMI** | **Flupirtin indication** | **Time to onset [days]** | **Dose [mg]** | **ALT  [µkat/l]** | **AST [µkat/l]** | **Symptoms** | **Medical history** |
| --- | --- | --- | --- | --- | --- | --- | --- | --- | --- | --- |
| **FL 1** | 51-60 | f | 23.8 | back pain / musculoskeletal disorders | 77 | 400 | 8.39 | 9.23 | Jaundice, fatigue | laparotomy and hematoma in the pelvis, repeated sectio caesarea |
| **FL 2** | 51-60 | f | 24.69 | back pain / musculoskeletal disorders | 70 | 200 | 12.14 | 3.45 | Jaundice, nausea, vomit | acute hepatitis, diagnosed 04/2013, anamnestic cured hepatitis B aged 11 years, chronic gastritis with helicobacter pylori eradication therapy, GIRD grade I, arterial hypertension |
| **FL 3** | 30-40 | f | no information | pain associated with multiple sclerosis | 23 | 200 | 52.74 | 35.36 | Jaundice, nausea, vomit | multiple sclerosis, retrobulbar neuritis, migraine, fatigue syndrome, ataxia, bladder dysfunction |
| **FL 4** | 71-80 | f | 31.25 | back pain / musculoskeletal disorders | 60 | 200 | 11.09 | 5.15 | Jaundice | histological findings of drug-induced hepatitis with high-dose pain medication (Ibuprofen and Katadolon; diagnosed 06/2010), arterial hypertension, hyperlipidemia, known Valoron allergy, diverse muscle-sceletal disorders, heart-valve disease, hypothyreosis/strumectomy, posterior vaginal wall carcinoma 1987, ovarial cyst removal 2008, glaucoma |
| **FL 5** | 71-80 | f | 23.83 | back pain / musculoskeletal disorders | 97 | 400 | 27.6 | 26.75 | Jaundice, nausea, vomit | liver cirrhosis, estrogen-induced hepatitis 1995, hypoalbuminemia, hepatic encephalopathy grade II, dehydration, ascites, hypocalcaemia with substitution, diverse drug therapy due to muscle-sceletal disorders |
| **FL 6** | 61-70 | f | 28.34 | back pain / musculoskeletal disorders | 52 | 200 | 36.59 | 24.58 | Jaundice | appendectomy 1959, cholecystectomy 1965, thyroidectomy 1965, nephropexy 1966, hysterectomy 1975, ablatio mammae 1976, herniated disc 2005, spinal stenosis 2005, chronic lower back pain |
| **FL 7** | 51-60 | f | 33.8 | back pain / musculoskeletal disorders | 92 | 400 | 41.03 | 21.64 | Jaundice | lumbar pain syndrome, hypothyroidism, arthritis |
| **FL 8** | 51-60 | f | 24.22 | back pain / musculoskeletal disorders | 11 | 400 | 14.67 | 14.76 | Jaundice, nausea, vomit | euthyroid metabolic condition without substitution, asthma bronchiale, Penicillin allergy, hypothyreosis/strumectomy 1998, osteochondrosis L4/5 & L5/S1 with Kissing-Spine-syndrome L4 to S1, coxa saltans after repeated hip surgeries |
| **FL 9** | 51-60 | f | 18.82 | back pain / musculoskeletal disorders | 90 | 400 | 1 | 1 | Jaundice | type I diabetes (diagnosed 1966) associated NASH-cirrhosis, chronic pain syndrome with arthrosis, omarthrosis right, spondylochondrosis, arterial hypertension, GIRD grade I, herpes zoster and hepatitis B infection 08/2011, portal-hypertensive gastropathy, ascites, facial paralysis 2002, Fissura Sylvii, diagnosed 2002, sectio caesarea 1982 |
| **FL 10** | 41-50 | f | 18.59 | back pain / musculoskeletal disorders | 74 | 200 | 13.12 | 10.58 | Jaundice, stomach pain | multiple suicide attempts, drug addiction (benzodiazepine), detoxification 2/2008, chronic nicotine abuse, alcohol abuse, benign essential hypertension |
| **FL 11** | 41-50 | f | 22.62 | back pain / musculoskeletal disorders | 23 | 400 | 52.44 | 27.06 | Jaundice | benign tumor in the left hepatic lobe, arterial hypertension, chronic pain syndrome with nucleus pulposus prolapse |
| **FL 12** | 51-60 | f | 26.03 | neuropathic pain after brain surgery (aneurysm) | 195 | 400 | 4.77 | 3.45 | Jaundice, fatigue, nausea | SMA positive 1:160, apparent abstinence from alcohol since 2008, aneurysm rupture with intracranial haemorrhage (surgery in 2006) |
| **FL 13** | 51-60 | f | 19.15 | back pain / musculoskeletal disorders | 15 | 600 | 1.09 | 0.83 | Jaundice | penicillin allergy |
| **FL 14** | 71-80 | m | 27.47 | polymyalgia rheumatica (PMR) | 21 | 400 | 1.94 | 1.57 | Elevated transaminases | polymyalgia rheumatica (PMR), diabetes mellitus (TDM2) |
| **FL 15** | 30-40 | f | 32.6 | back pain / musculoskeletal disorders | 32 | 400 | 35.2 | 14.47 | Jaundice, fatigue, pruritus | back pain |

**References**

1. Guo J, Loke J, Zheng F, et al. Functional linkage of cirrhosis-predictive single nucleotide polymorphisms of toll-like receptor 4 to hepatic stellate cell responses. Hepatology 2009;49:960-8.

2. Lu X, Ji C, Tong W, et al. Integrated analysis of microRNA and mRNA expression profiles highlights the complex and dynamic behavior of toosendanin-induced liver injury in mice. Sci Rep 2016;6:34225.
